# Ultra-Stretchable Anti-Freezing Hydrogel Electrolytes Cross-Linked by Liquid Metal Particle Initiators Toward Soft Energy Storage Devices

**DOI:** 10.1007/s40820-026-02126-7

**Published:** 2026-03-13

**Authors:** Qingshi Zhang, Priyanuj Bhuyan, Que Thi Nguyen, Xia Sun, Kunlong Liang, Mukesh Singh, Subir Kumar Pati, Xianglan Li, Yeeshu Kumar, Sungjune Park

**Affiliations:** 1 School of Chemical Engineering, Sungkyunkwan University (SKKU), Suwon, 16419 Republic of Korea; 2 Wearable Fluidic, Inc, Suwon, 16419 Republic of Korea; 3 Sustainable Functional Biomaterials Laboratory, Bioproducts Institute, Department of Wood Science, Faculty of Forestry, University of British Columbia, Vancouver, BC V6T 1Z4 Canada

**Keywords:** Liquid metal nanoparticle initiator, Anti-freezing hydrogel, Wearable device, Supercapacitor

## Abstract

Hydrogels are appealing for electrolyte of soft energy storage devices due to their mechanical flexibility and high ionic conductivity. Their polymeric networks containing large amount of water result in mechanical flexibility suitable for soft devices. However, high water content in hydrogels results in insufficient mechanical strength and freezing at sub-zero temperatures. Herein, we developed anti-freezing hydrogels with high mechanical strength by liquid metal (LM)-initiated free-radical polymerization. During the polymerization, we introduced stearyl methacrylate (SMA) to form hydrophobic associations, increasing the physical cross-linking density within the polymer network, resulting in desirable mechanical properties (elongation at break of 907% and tensile strength of 766 kPa). The hydrogel exhibiting ionic conductivity of 4.35 S m⁻^1^ at 25 °C after immersing in LiCl solution showed the slightly decreased ionic conductivity (3.39 S m⁻^1^) and almost maintained mechanical stretchability (elongation at break of 897%) after being stored at − 20 °C for 12 h. Supercapacitors consisted of the hydrogel electrolyte and activated carbon electrodes achieved a high areal capacitance (93.52 mF cm⁻^2^) due to rapid ionic mobility through the hydrogel electrolyte and retained 98% of its capacitance after 45,000 charge–discharge cycles due to enhanced polymeric network of electrolyte via LM particles-initiated polymerization and SMA-induced hydrophobic association.

**Supplementary Information:**

The online version contains supplementary material available at 10.1007/s40820-026-02126-7.

## Highlights

- Robust hydrogel electrolytes derived from liquid metal-initiated polymerization and increased hydrophobic association.
- Anti-freezing hydrogels achieved by disrupting hydrogen bonds between water molecules.
- Hydrogel electrolyte-enabled supercapacitors achieving high performance and mechanical deformability.

**Supplementary Information:**

The online version contains supplementary material available at 10.1007/s40820-026-02126-7.

## Graphical abstract

**Figure:**
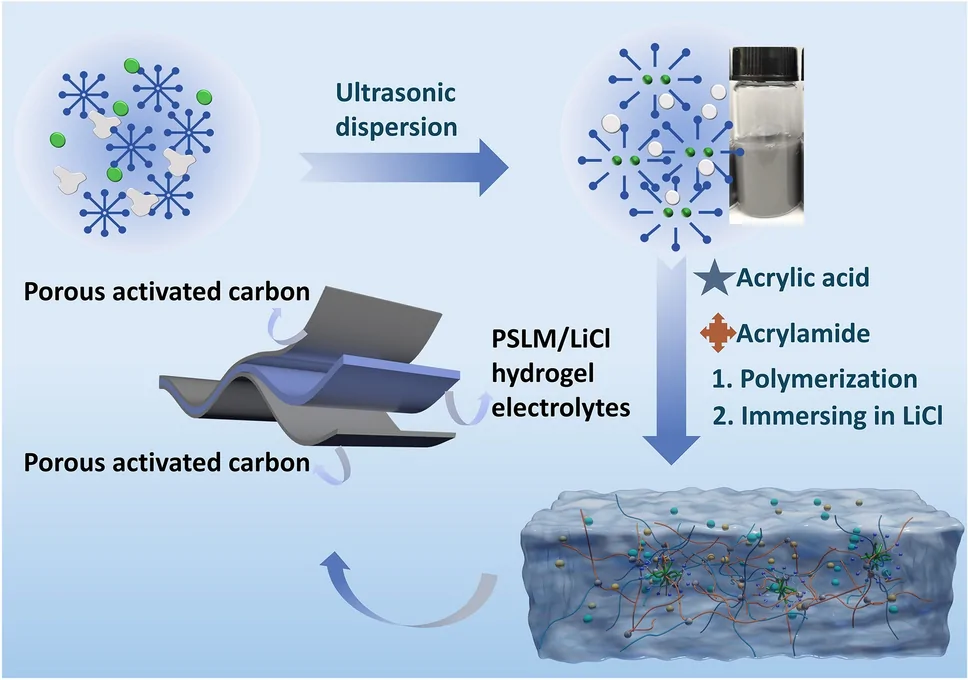

## Introduction

As energy demand for next-generation portable and wearable electronics intensifies, soft and stretchable supercapacitors have emerged as promising energy storage solutions. They offer a unique synergy of high-power density, fast charging-discharging ability and durability [1–3]. Their engineered flexibility and mechanical compliance further aids integration them onto conformal and deformable electronics systems. Supercapacitor comprises of two main components: a set of electrodes responsible for charge accumulation and a separator for physically isolating the electrodes and facilitating ionic transport [4–7]. The use of liquid electrolytes, such as aqueous or organic electrolytes is advantageous due to their rapid ionic mobility [8], however, the liquid electrolytes can raise safety issues such as thermal instability-induced decomposition, flammability of organic solvents, high probability of leakage. In particular, they lack mechanical strength and integrity under deformation when it comes to their direct use in wearable device [9]. Solid electrolytes providing mechanical flexibility can address these issues; however, they are limited by insufficient ionic mobility, limited voltage window and high internal resistance, thereby lowering the energy storage ability [10, 11]. These challenges highlight the continuing need for developing electrolyte materials that effectively facilitates ionic mobility, leakage resistance, mechanical adaptability for soft energy storage devices.

Hydrogels are promising materials that can address these issues of electrolytic separators [12]. They can facilitate high ionic conductivity and can be operated at high voltage window along with mechanical flexibility. Hydrogels are accompanied by unbound or free water, i.e., the water in the 3D polymeric network that are not strongly associated with the polymer [13–15]. Although this unbound water is responsible for ionic transport due to its high mobility, they are susceptible to freezing. Freezing alters the polymeric structures by forming crystals and immobilizes the ions, thereby reducing the ionic conductivity, as well as deteriorating mechanical softness [16]. Therefore, freezing behavior poses significant challenges for practical deployment of hydrogel-based electrolytes in energy storage devices in sub-zero temperature environments where energy generation and storage become more challenging due to reduced electrochemical performances [17]. To prevent freezing behavior of the hydrogels, organic solvents such as ethylene glycol and glycerol have been introduced into hydrogels [18, 19]. While these approaches can utilize the high density of hydrogen bonding between solvent and water molecules, resulting in lowering the freezing point and maintaining electrochemical performances even at − 40 °C, they still face insufficient mechanical strength [20]. The incorporation of hydrophilic inorganic salts, such as LiCl, can also reduce the freezing point by disrupting water hydrogen bonding [21]. The relatively small Li⁺ ions enable them to strongly interact with water molecules, thereby immobilizing them within the hydrogel matrix, resulting in lowering the freezing point. The LiCl hydrogel is operable at wide voltage window and exhibits increased mechanical strength and flexibility, thus providing stable and high electrochemical performances maintained while they are strained.

**Fig. 1:**
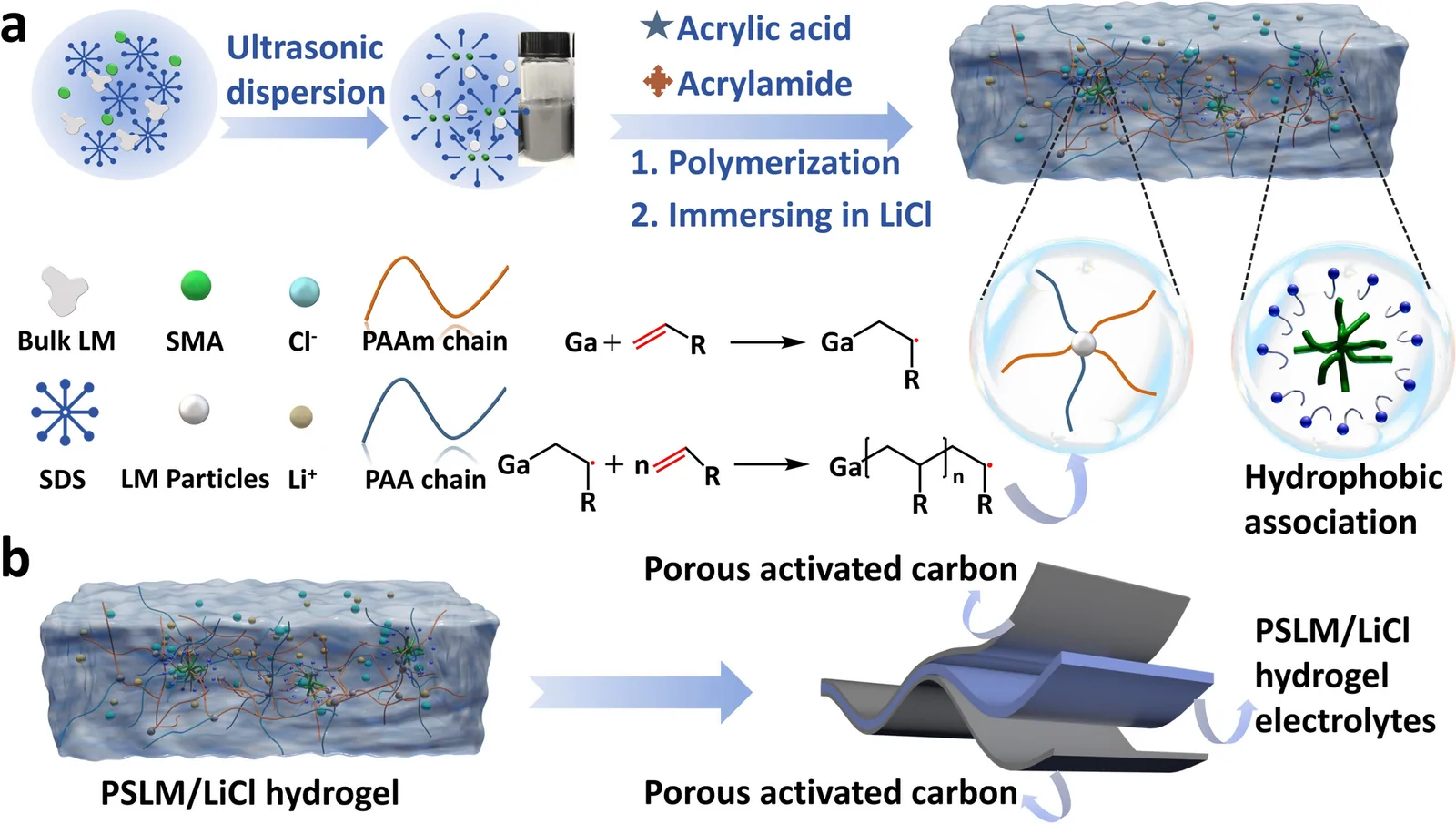
**a** Schematic illustration of preparation of PSLM/LiCl hydrogel. **b** Schematic showing structure of supercapacitor including PSLM/LiCl hydrogel electrolyte between porous activated carbon electrodes

Recently, liquid metals (LMs) have attracted great interest for applications in soft and stretchable electronics due to their metallic conductivity even while strained [22–27]. They form native oxide layer on the surface spontaneously, therefore, they can adhere on any substrates and pattern into desired geometries by various unconventional approaches [28–31]. The LMs can form small-sized particles by applying shear force and ultrasonication due to the fluidic nature of the metals [32]. While the LM particles-dispersed elastomers exhibiting unique features in terms of mechanical, electrochemical and thermal properties have been extensively explored [33–36], the LM particles-incorporated hydrogels have relatively been less studied due to low interfacial compatibility between the LM particles and hydrogels. Recently, the LM particles have been used as initiators to polymerize vinyl monomers that can form physical gels [37, 38]. An oxide layer formed on the metal isolates the metal from the solution and the small particles created by ultrasonication increase the surface area of the metal, resulting in rapid polymerization. While conventional molecular initiators require external sources such as thermal or photo energy, the LM nanoparticles exposed to the solution initiate the free-radical polymerization by reaction between unpaired electrons of gallium and π bonds of the vinyl monomers [39]. In this work, we also developed liquid metal particles-incorporated hydrogel by a same principle. The hydrogel is polymerized by liquid metal particles initiator within 1 min under ultrasonication due to large surface area of the metal via formation of liquid metal nanoparticles, but stearyl methacrylate (SMA) is newly introduced to form hydrophobic associations within the polymeric network, resulting in increased density of physical cross-linking. Therefore, remarkably, less liquid metal initiator was used for polymerization than in previous studies [38]. Once polymerized, we immersed the hydrogel into deionized (DI) water containing LiCl that can achieve ionic conductivity of 4.35 S m^−^^1^ at 25 °C and effectively destroy the hydrogen bonding between water molecules, thereby lowering the freezing point to below-40 °C. This anti-freezing ability of LiCl-immersed hydrogel allows to have excellent ionic conductivity (~ 3.39 S m^−^^1^) and high stretchability (897%) even after storage at −20 °C for 12 h. We further used the liquid metal nanoparticles-initiated hydrogel as electrolytes of soft and stretchable supercapacitor **(**Fig. 1b**)**, achieving high area capacitance (93.52 mF cm^−2^), superb energy storage capacity (energy density of 12.9 µWh cm^−2^ and power density of 400 µW cm^−2^), and large deformability. This approach to fabricate LM particles-utilized hydrogel electrolytes would be further used for wearable energy storage and self-powered devices.

## Experimental Section

### Materials

Acrylamide (AM), sodium dodecyl sulfate (SDS), lithium chloride (LiCl), sodium chloride (NaCl), 1-methyl-2-pyrrolidone (NMP) and poly(vinylidene fluoride) (PVDF) were purchased from Sigma-Aldrich (Germany). Acrylic acid (AA) were purchased from SAMCHUN (Korea). Stearyl methacrylate (SMA) were purchased from TCI (Japan). Liquid Metal (Gallium–Indium Alloy, EGaIn) were purchased from Indium Corporation (USA). Multi-walled carbon nanotubes (MWCNTs) and graphite nanofibers (GNF) were purchased from Carbon nano-material Technology (Korea). Carbon black (C-65) were purchased from MTI Korea. Porous activated carbon were purchased from Power Carbon Technology (Korea).

### Preparation of PSLM/LiCl Hydrogel

The PSLM/LiCl hydrogel was synthesized via free-radical polymerization using AM and AA as monomers, SMA as a physical cross-linker, and liquid metal as the initiator. After gelation, the hydrogel was immersed in DI water with various concentrations of LiCl to obtain the conductive PSLM/LiCl hydrogel. First, SMA, SDS, and NaCl were dissolved in DI water. Subsequently, varying amounts of liquid metal were added to the solution, followed by ultrasonication for 5 min to obtain a uniform dispersion. AM and AA monomers were then added to the solution and dissolved completely, followed by ultrasonication for 50 s. The resulting homogeneous mixture was poured into a hydrogel mold and became gel by free-radical polymerization for 1 min. After gelation, the hydrogel was removed from the mold and immersed in LiCl solutions of different concentrations to yield the conductive PSLM/LiCl hydrogels.

### Assembly of Supercapacitor Devices

#### Electrode Preparation

Three types of active materials, including porous activated carbon, MWCNTs and graphite nanofibers, were used to fabricate electrodes. For each electrode, the active material was mixed with carbon black and PVDF in a weight ratio of 8:1:1, and dispersed in an appropriate amount of NMP to form a homogeneous slurry. The slurry was uniformly coated onto a 1 cm x 1 cm carbon cloth and dried in an oven at 60 °C for 8 h. The mass loading for each electrode was approximately 1.6 mg.

#### Assembly of the Device

Two active porous carbon electrodes with equal mass loading deposited on carbon cloth were placed on both sides of the PSLM/LiCl hydrogel electrolyte to assemble a solid-state supercapacitor in a sandwich-like configuration. For cycling stability tests and serial connection of supercapacitors, electrodes with a size of 1 cm × 2 cm were used, with a mass loading of approximately 2.5 mg cm^−^^2^. For bending tests, larger electrodes with a size of 1 cm × 3 cm were employed to facilitate mechanical deformation during measurement.

### Characterization

#### Mechanical Properties

Mechanical properties were tested using a universal mechanical testing instrument (Quasar 2.5 single column). The samples were prepared in a standard dog-bone shape with a thickness of 2 mm. The tensile tests were conducted at a strain rate of 50 mm min^−^^1^, while loading–unloading cyclic tests were performed at a strain rate of 200 mm min^−^^1^. Unless otherwise specified, tensile tests were performed on hydrogels after LiCl immersion and equilibration; mechanical data of freshly prepared hydrogels before LiCl immersion are provided separately in Fig. S4.

#### Measurement of Conductivity of PSLM/LiCl Hydrogel Electrolyte

Ionic conductivity (σ) was measured using electrochemical impedance (EIS) with a VersaSTAT3 (Ametek, USA) electrochemical workstation. Hydrogel samples were immersed in LiCl solutions of various concentrations for 1 h. After immersing, the length, width, and thickness of each sample were measured. During characterization, copper tape was directly applied to the top and bottom surfaces of the hydrogel, serving as electrodes for the impedance measurements. For each concentration, five samples were tested. The highest and lowest resistance values were excluded, and the average of the remaining three was used. The ionic conductivity (σ, S m^−^^1^) is calculated using Eq. (1):

1 $${\upsigma }\,{ = }\,L\,/\,\left( {R\, \times \,A} \right)$$

where *R* is the measured resistance, *A* is the contact area between the hydrogel and electrodes, and *L* is the hydrogel thickness.

#### Morphological and Functional Characterization

The Fourier-transform infrared (FTIR) spectra of the hydrogels were recorded over the wavenumber range of 400–4000 cm^−^^1^ using an FTIR spectrometer (FT/IR-4x, JASCO). Raman spectra were acquired in the range of 400–3500 cm^−^^1^ using a Raman spectrometer (LabRam Soleil, HORIBA). The antifreeze behavior of the hydrogel was evaluated by differential scanning calorimetry (Nexta DSC 600, Hitachi) over a temperature range from − 50 to 25 °C to analyze the phase transition behavior. Rheological measurements were conducted using a rheometer (ARES-G2, TA Instruments). To assess the antifreeze properties, temperature-dependent rheology was performed from − 50 to 25 °C. In addition, oscillatory strain sweep tests were carried out in the strain range of 0.01% to 100% to evaluate the viscoelastic behavior of the hydrogel. The internal microstructure of freeze-dried hydrogels was observed using a scanning electron microscope (SEM, JSM-7600F, JEOL).

#### Electrochemical Characterization of Supercapacitors Devices

Cyclic voltammetry (CV), galvanostatic charge–discharge (GCD), and electrochemical impedance spectroscopy (EIS) were performed using a VersaSTAT3 electrochemical workstation (Ametek, USA). Cycling stability was assessed by repeated GCD measurements over 45,000 charge–discharge cycles. The areal capacitance of the single electrode (C_sp_, mF cm^−2^) was calculated from the GCD discharge curves using Eq. (2) [40]:

2 $$C_{{{\mathrm{sp}}}} = 4\frac{I\Delta t}{{A\Delta V}}$$

where I is the discharge current (mA), Δ*t* is the discharge time (s), ΔV is the potential window (V), and *A* is the area of the electrode (cm^2^).

The areal energy density (E, µWh cm^−^^2^) and power density (P, µW cm^−^^2^) were calculated as follows:

3 $$E = \frac{{C_{sp} \times \left( {\Delta V} \right)^{2} }}{7200}$$

4 $$P = \frac{E \cdot 3600}{{\Delta t}}$$

where E is the energy density and *P* is the power density, with Δt taken from the GCD discharge time.

#### Statistical Analysis

The data with error bars is presented as mean ± standard deviation, calculated from at least three independent samples.

## Results and Discussion

### Fabrication of PSLM/LiCl Hydrogel

Figure 1a shows a schematic diagram of preparing the polyacrylamide/poly(acrylic acid)/stearyl methacrylate/liquid metal/lithium chloride (PSLM/LiCl) hydrogel featuring interpenetrating network structures. To prepare the hydrogel, sodium chloride (NaCl), stearyl methacrylate (SMA), and sodium dodecyl sulfate (SDS) were dissolved in water. Various amounts of LM were then added to the solution and sonicated to form LM nanoparticles and SMA was dispersed in the solution with the help of SDS. Acrylamide (AM) and acrylic acid (AA) monomers were added and sonicated to allow SMA to be copolymerized with AM and AA monomers. The SMA hydrophobic segments within the SDS micelles aggregate to form hydrophobic associations, which act as physical cross-linking points, thereby increasing the cross-linking density of the hydrogel [41]. The polymerization is initiated by LM. Under ultrasonication, the oxide layer on the LM surface undergoes continuous disruption and regeneration, which exposes gallium atoms with unpaired electrons. These liquid metal particles can directly interact with the π bonds of vinyl monomers. Ga^3^⁺ ions released from the LM surface interact with carboxyl groups on PAA chains through ionic coordination, contributing to physical cross-linking within the hydrogel network (Fig. S1). As shown in Fig. S2, different amounts of LM (0.05, 0.075, and 0.1 g) were used to explore the optimized dosage of LM initiators and evaluate their effect on hydrogel formation and structure. The results showed that with increasing LM content, the hydrogel surface became progressively rougher, and LM aggregation appeared within the hydrogel presumably due to excessive LM caused local aggregation, which disrupted the formation and integrity of the hydrogel network. As shown in Fig. S3a, the hydrogel undergoes rapid gelation within 1 min at room temperature upon uniform dispersion of LM into the monomer solution. This observation confirms that LM acts as an efficient radical initiator, enabling fast polymerization without any photo- and thermal initiators. The resulting hydrogel exhibits mechanical deformability as shown in Fig. S3b, highlighting its potential for application in soft devices. We further characterized the mechanical properties of hydrogels containing different amounts of LM. As shown in Fig. S4a, the hydrogel with 0.05 g LM exhibited the highest elongation at break (1254%), due to less amount of oxides on the LM particles. Young’s modulus of the hydrogels is slightly increased with higher LM content (Fig. S4b) due to increased surface area of the oxide and viscosity as a function of LM ratio. Therefore, we chosen the hydrogel containing 0.05 g of LM for further characterizations and applications in hydrogel electrolytes for soft energy storage devices, i.e., supercapacitors as shown in Fig. 1b.

### Characterization of PSLM/LiCl Hydrogel

**Fig. 2:**
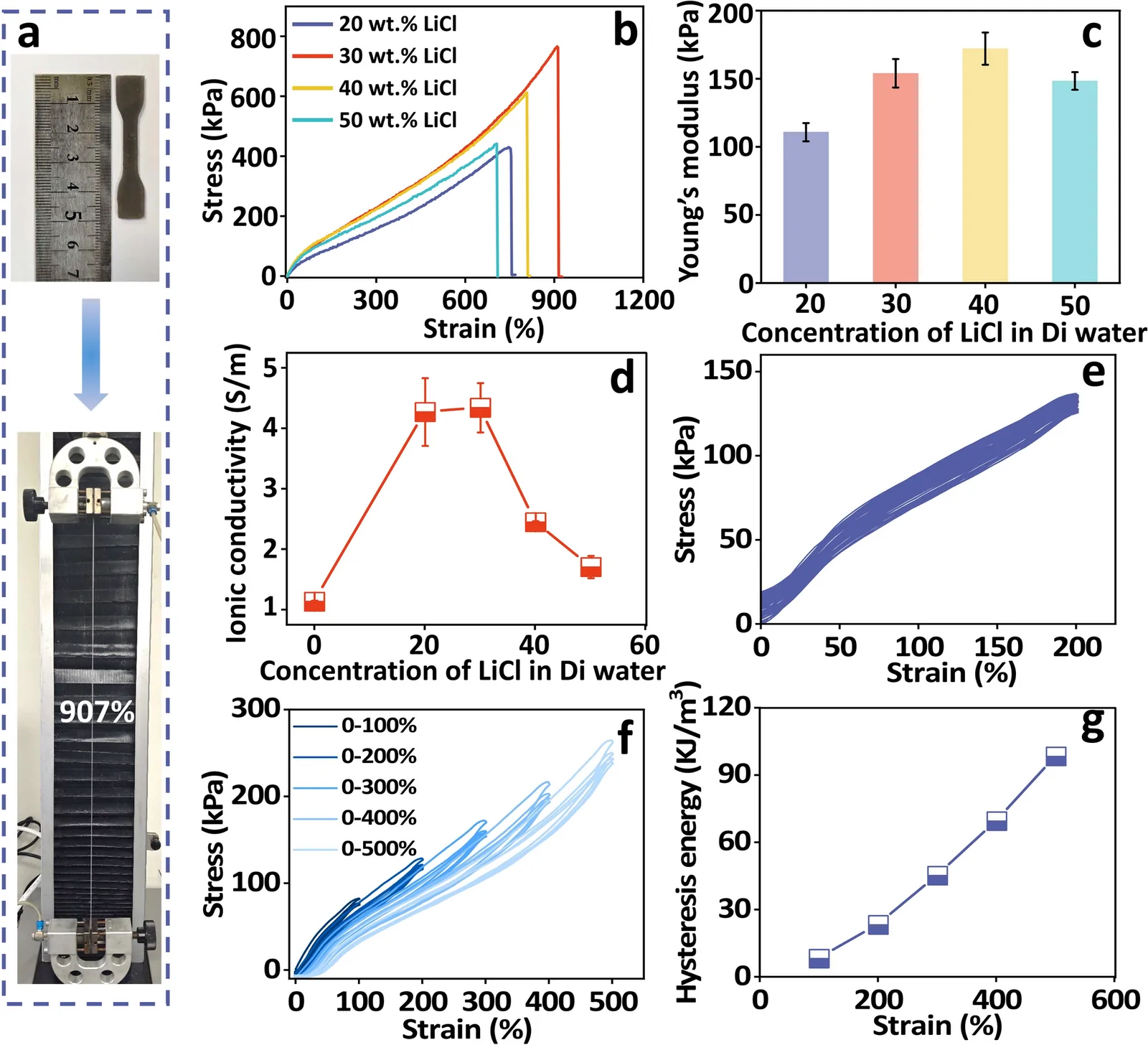
Mechanical characterization of PSLM/LiCl hydrogels. **a** Photograph showing hydrogel during tensile test after immersing in DI water containing 30 wt% of LiCl (PSLM/LiCl). **b** Tensile stress–strain curves of hydrogels after immersing in DI water containing various concentrations of LiCl (20–50 wt%). **c** Young’s modulus and **d** ionic conductivity of hydrogels after immersing in DI water containing various concentrations of LiCl (0–50 wt%). **e** Stress–strain curves of PSLM/LiCl hydrogels during 50 consecutive cycles of tensile strains with a maximum strain of 200%. **f** Stress–strain curves of PSLM/LiCl hydrogels during 4 consecutive cycles of various tensile strains (100%–500%) and **g** Hysteresis energy of PSLM/LiCl hydrogels as a function of tensile strains (100%–500%)

Hydrophobic associations from SMA segments and ionic coordination between Ga^3^⁺ and carboxyl groups of PAA chains allow the hydrogel to increase cross-linking density, thereby imparting the enhanced mechanical strength. While the SMA-non containing sample is highly viscous due to less cross-linking density in the polymeric network as shown in Video S1. The SMA-containing hydrogel showed markedly enhanced mechanical strength, reaching a tensile stress of 796 kPa and elongation at break of 1254% (Fig. S4a). This result indicates that the hydrophobic associations formed by SMA segments effectively contribute to improved mechanical strength by acting as physical cross-linking points in the polymeric network. As shown in Fig. 2b, the stress versus strains of the hydrogels after immersing in DI water containing various concentration of LiCl. As the LiCl concentration increased from 20 to 30 wt%, both the elongation at break and tensile strength showed an upward trend, with the tensile strength of 766 kPa and elongation at break of 907% at 30 wt%. However, further increasing the LiCl concentration 50 wt% resulted in decreased the tensile strength and elongation at break presumably due to excessive ionic concentration-induced disrupting the hydrogen bonding in the polymeric (Fig. 2c).

Figure S5 shows the equivalent series resistance of the hydrogels in the high-frequency range with increasing ionic concentration. Overall, the ionic conductivity is increased as a function of LiCl contents, reaching a maximum of 4.35 S m^−1^ at 30 wt% (Fig. 2d). This increase is mainly attributed to the increased concentration of mobile ions. However, when the LiCl content exceeds 30 wt%, the ionic conductivity gradually decreases. This decline can be ascribed to the formation of ion pairs or ion clusters at high salt concentrations, which reduces the number of free ions. In addition, excessive LiCl strongly coordinates with water molecules, decreasing the amount of free water and suppressing ion mobility, thereby leading to reduced ionic conductivity at higher LiCl contents. Considering the mechanical properties and ion conductivity, we further characterized and utilized the hydrogel formed by using 0.05 g of LM initiators after immersing in DI water containing 30 wt% of LiCl. The stress–strain curves of the hydrogel after immersing in DI water containing 30 wt% of LiCl shows high resilience (i.e., the ratio of energy absorbed during loading to energy released during unloading) during 50 consecutive cycles of tensile strain until a maximum strain of 200%, indicating slightly dissipated energy during strain cycles, i.e., low hysteresis energy (Fig. 2e). Furthermore, Fig. S6 shows that hysteresis energy of the hydrogel during the first strain is relatively high (> 26.40 kJ m^−3^) due to the Mullins effect; however, it decreases and stabilizes at around 10 kJ m^−3^, signifying the stable mechanical response during cyclic tensile deformation of the hydrogel. Figure 2f further shows the stress versus cyclic strains of the hydrogel at different maximum strains (100%–500%). With increasing strain, the hydrogel exhibits increased hysteresis energy during strain cycles presumably due to loose entangled polymeric network as a function of strain (Fig. 2g**)**.

**Fig. 3:**
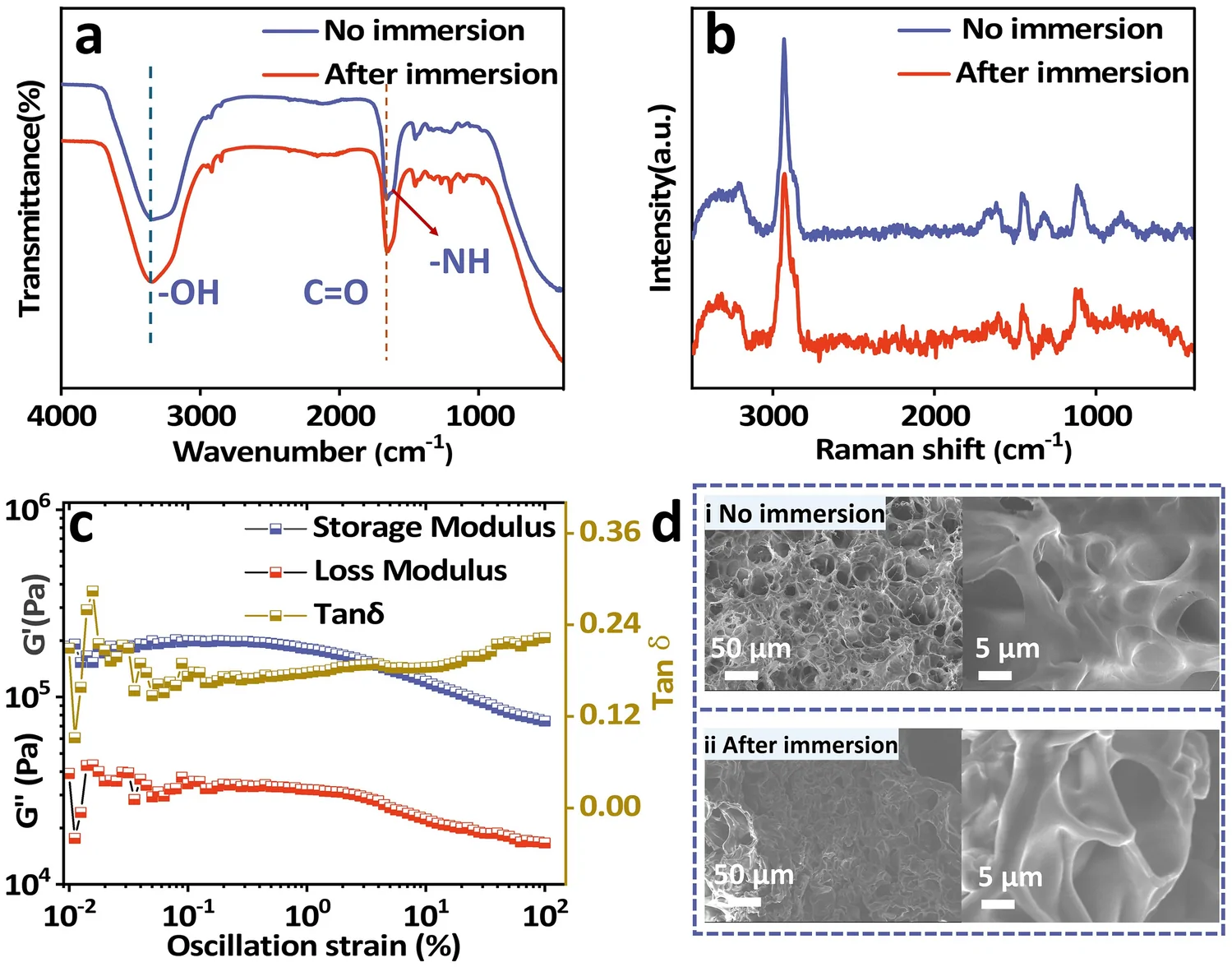
**a** FTIR, **b** Raman spectra of hydrogel before- and after immersing in DI water containing 30 wt% of LiCl. **c** Oscillatory strain sweep measurements of hydrogel after immersing in DI water containing 30 wt% of LiCl. **d** SEM images of the hydrogel before- and after immersing in DI water containing 30 wt% LiCl

To further characterize the internal structure and physicochemical properties of the hydrogel, a series of morphological and spectroscopic analyses were carried out. To investigate the structural evolution induced by immersing in LiCl, FTIR spectra of the hydrogel before and after LiCl treatment were recorded, as shown in Fig. 3a. After immersion of the hydrogel in DI water containing 30 wt% of LiCl, the hydrogel shows broad –OH stretching band (~ 3329 cm^−^^1^) exhibiting a slight blue shift, moving toward higher wavenumbers. This shift indicates a partial disruption of the hydrogen bonding network between water molecules within the hydrogel, likely due to the strong ionic hydration of Li⁺. The formation of Li⁺·(H_2_O)_n_ hydration complexes reduces the extent of inter- and intramolecular hydrogen bonding, thereby increasing the O–H vibrational frequency [42]. Notably, the sharp –NH bending vibration observed at ~ 1610 cm^−^^1^ in the pristine hydrogel nearly disappears after LiCl treatment. This change may be attributed to the interaction between Li⁺ ions and the amide groups, together with the disruption of hydrogen bonding within the hydrogel network [43]. The Raman profiles exhibit no significant shift in peak positions and appearance of new bands before- and after LiCl treatment, indicating that the backbone structure of the hydrogel remains chemically stable (Fig. 3b). The characteristic vibrational modes corresponding to C–C and C–N stretching remain unchanged, suggesting that Li⁺ does not induce chain scission or chemical rearrangement within the polymer matrix. As shown in Fig. 3c, the viscoelastic behavior of the LiCl-immersed hydrogel was evaluated via oscillatory strain sweep measurements. The storage modulus (G’) remains consistently higher than the loss modulus (G’’) across the entire strain range (0.01%–100%), indicating that the hydrogel exhibits elastic-dominant behavior characteristic of a solid-like material. As the strain increases beyond 1%, G’ and G’’ begin to decrease slightly, accompanied by a gradual rise in the loss factor (tanδ), suggesting the onset of network softening due to partial disruption of physical cross-links. Nevertheless, tanδ remains below 0.24 throughout the entire measurement range, confirming that the hydrogel retains its elastic character even under large deformations. As shown in Fig. 3d, while the pore size became significantly smaller and the network gets more compact after immersion in LiCl solution, the immersed hydrogels ensure continuous ionic pathways, supporting the ionic conductivity results as shown in Fig. 2d**.**

### Anti-Freezing Properties of PSLM/LiCl Hydrogel

**Fig. 4:**
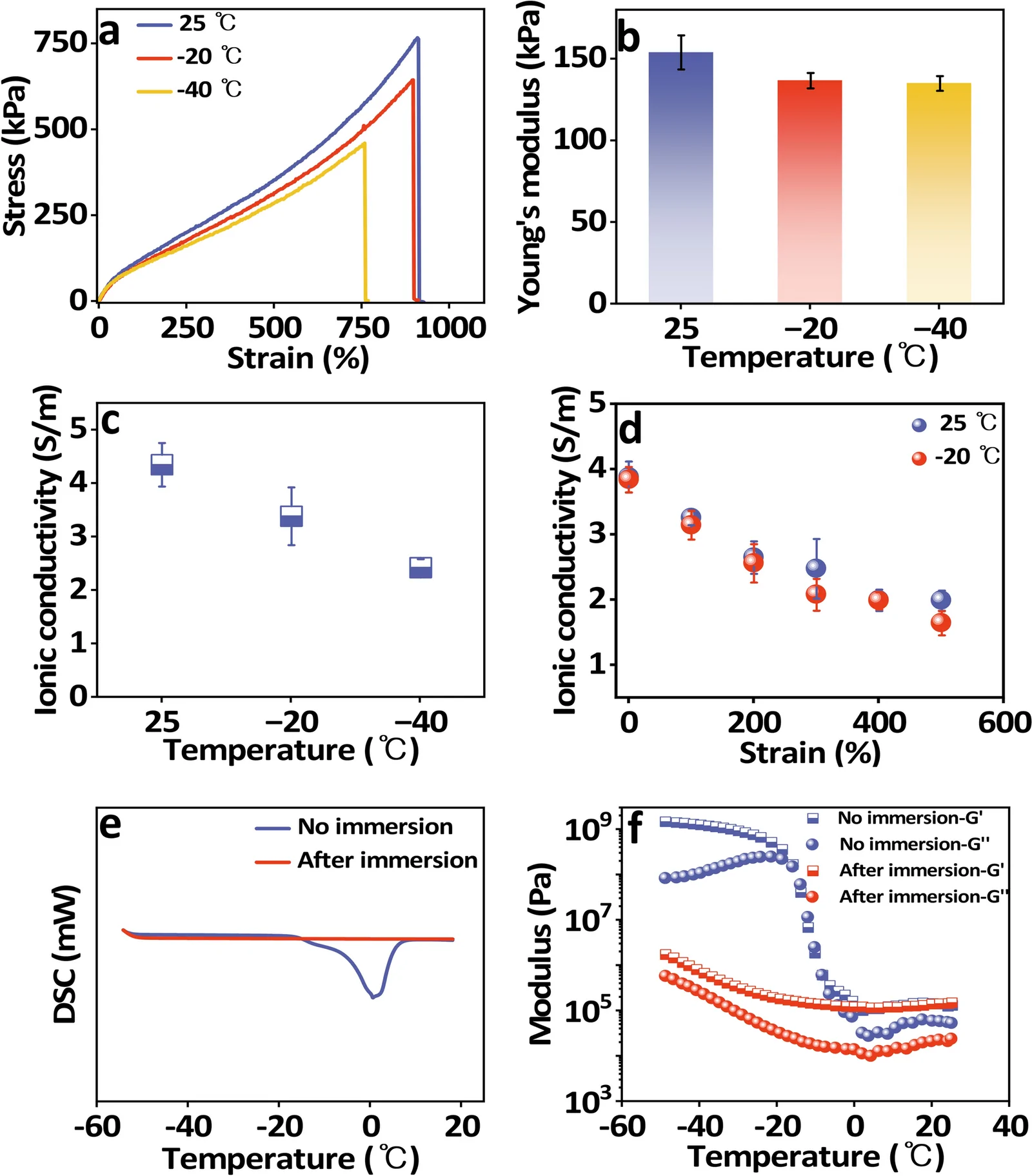
Anti-freezing properties of PSLM/LiCl hydrogels. **a** Tensile stress–strain curves and **b** Young’s modulus of PSLM/LiCl hydrogels measured after storage at various temperatures ranging from − 40 to 25 °C for 12 h. **c** Ionic conductivity of PSLM/LiCl hydrogels after storage at temperatures ranging from − 40 to 25 °C for 12 h. **d** Ionic conductivity of PSLM/LiCl hydrogels measured at various tensile strains (0–500%) after storage at two temperatures (25 and − 20 °C) for 12 h. **e–f** DSC and rheological curves of hydrogels before and after immersing in DI water containing 30 wt% of LiCl

Hydrogels typically contain two main types of water molecules: bound water and free water. Bound water molecules form strong hydrogen bonds with polymer chains or ions and do not contribute to crystallize even at sub-zero temperatures. However, free water molecules behave similarly to bulk water, thus crystallize the hydrogel network near 0 °C [16]. The introduction of hydrophilic salts such as LiCl enables the stable hydration with water molecules. This interaction reduces the amount of free water and depresses the freezing point, thereby imparting the hydrogel with excellent antifreeze capability and high ionic conductivity even at sub-zero temperatures. Figure 4a shows the tensile stress–strain curves of the LiCl-immersed hydrogels after storage at 25, − 20, and − 40 °C for 12 h. Both tensile strength and elongation at break decrease with decreasing storage temperature, which is mainly related to the water dominated nature and dynamic network structure of the hydrogel. DSC results confirm that water remains in a non-freezable, strongly bound state at low temperatures, continuously plasticizing the polymer network. Meanwhile, the hydrogel is primarily physically cross-linked, and reduced temperature weakens dynamic interactions within the network, leading to a decreased effective load-bearing capacity. Nevertheless, the hydrogel can deform above 700% of strain even after storage at − 40 °C, indicating high deformation property preserved even at sub-zero temperature conditions. Young’s modulus of the hydrogel after immersing in DI water containing 30 wt% of LiCl decreases with decreasing storage temperature **(**Fig. 4b**)**. To further verify the mechanical reliability of the LiCl-immersed hydrogels at low-temperature conditions, cyclic tensile tests were performed after storage at − 20 °C for 12 h (Fig. S7). The cyclic tensile tests at different maximum strains (100%–500%) showed stable loading–unloading behavior except in the first cycle showing relatively large hysteresis energy due to the Mullins effect. This result confirms that the LiCl-immersed hydrogel after storage at − 20 °C preserves excellent resilience over a wide deformation range. Figure 4c shows the ionic conductivity of the hydrogel after immersing in DI water containing 30 wt% of LiCl and storage at temperatures ranging from 25 to 40 °C. Although the ionic conductivity of the hydrogels decreases as the temperatures decreases, they exhibit good ion transport capability even after storage at sub-zero temperature conditions. We note that conductivity of the hydrogels at sub-zero temperature is attributed to the fact of that Li⁺ and Cl⁻ ions form stable hydration within the hydrogel network, thereby effectively suppressing the crystallization of free water. We also characterized the conductivity (σ) of the LiCl-immersed hydrogels upon mechanical deformation over a strain range of up to 500%. During the measurement, the hydrogel sample was clamped between two copper tape electrodes for electrochemical impedance spectroscopy (EIS) analysis to determine its bulk resistance (R). Throughout the measurements, the contact area (A) between the electrodes and the hydrogel was kept constant at 1 cm^2^, and the sample thickness (L) was recorded at each strain level. The ionic conductivity (σ) was calculated using the equation σ = L/(R × A). Unlike electronic conductors, the hydrogel electrolyte exhibited nearly constant resistance under mechanical deformation, indicating stable ion transport properties. This behavior was observed consistently at both 25 and − 20 °C (Fig. S8). As shown in Fig. 4d, the ionic conductivity of the hydrogel initially decreases with increasing strain and stabilizes at around 2.5 S m^−^^1^ due to the reduction in film thickness. However, even when the strain exceeds 500%, the conductivity retains more than 50% of its original value, suggesting that the hydrogel network provide continuous ion transport pathways even upon high degree of stains. Figure 4e shows a distinct endothermic peak corresponding to free water at approximately 0.7 °C in the non-immersed hydrogel, whereas no obvious free water signal is observed in the LiCl-immersed sample, indicating that free water is largely eliminated, likely due to strong hydration between Li⁺ and water molecules. Complementary rheological analysis further supports this observation. In Fig. 4f, the non-immersed hydrogel exhibits a sharp increase in both G’ and G’’ near 0 °C , indicating freezing-induced stiffening of the hydrogel network. In contrast, the LiCl-immersed hydrogel maintains stable G’ and G’’ values even as the temperature decreases, suggesting that the LiCl incorporation effectively suppresses ice formation within the polymer matrix. These results clearly confirm that LiCl treatment can preserve the mechanical flexibility of the hydrogel at sub-zero temperature conditions. Figure S9a, b shows the deformability and conductivity of the hydrogels after storage at − 20 °C. While the non-immersed hydrogel becomes stiff due to freezing internal water at − 20 °C, the LiCl-immersed hydrogel remains stretchable after storage at − 20 °C, demonstrating its anti-freezing feature. The non-immersed hydrogel produced only a faint luminescence after storage at − 20 °C, indicating that ice formation severely impeded ion transport. In contrast, the LiCl-immersed hydrogel illuminated the LED brightly after storage at − 20 °C, providing a direct visual demonstration that LiCl suppresses free water crystallization, thereby maintaining continuous ionic pathways for efficient charge transport even at sub-zero temperature conditions.

### Electrochemical Performance of Supercapacitors with PSLM/LiCl Hydrogel Electrolytes

**Fig. 5:**
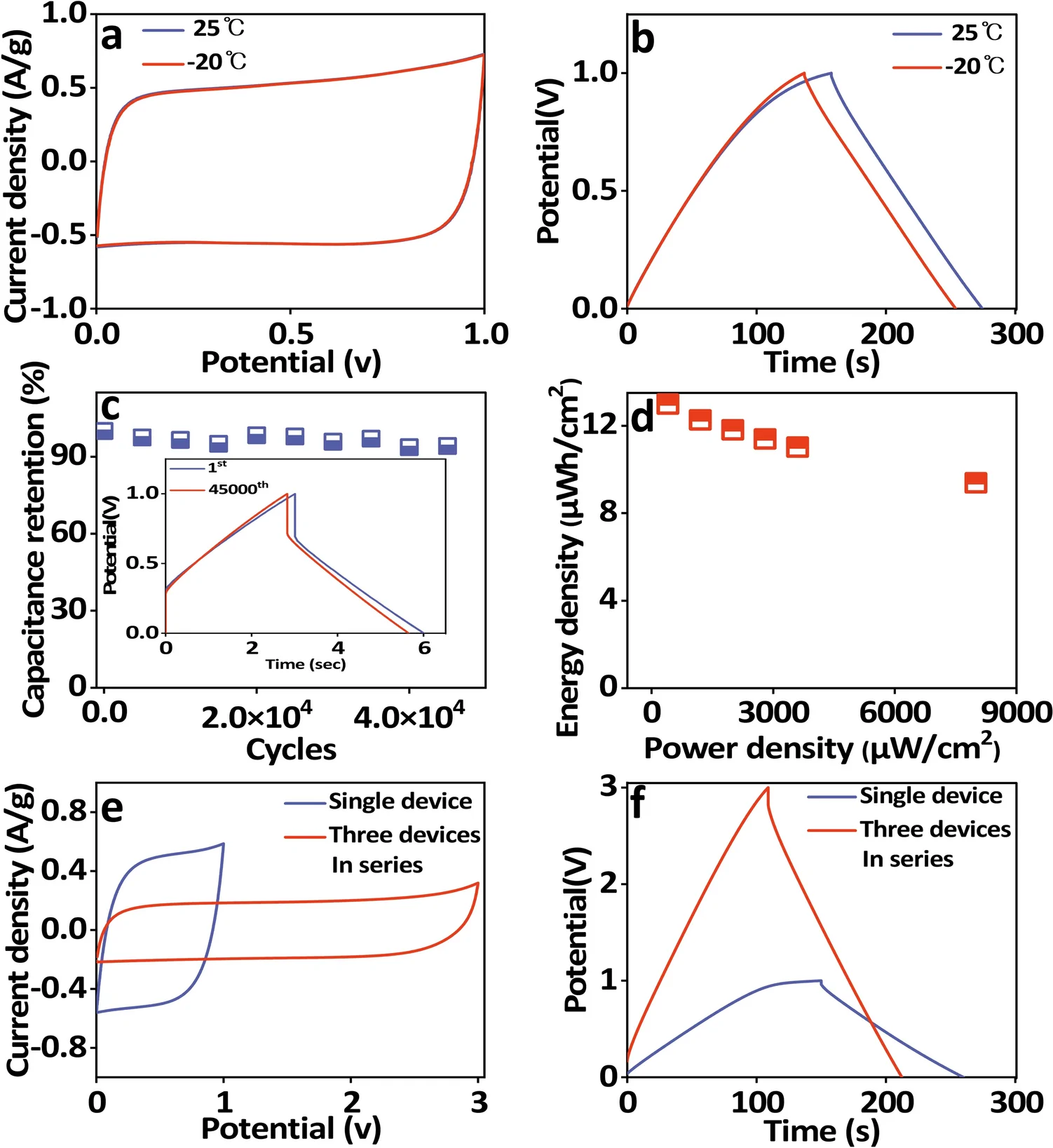
**a** CV curves of supercapacitors with PSLM/LiCl electrolyte and PAC electrodes measured at a scan rate of 50 mV s⁻^1^ after storage at 25 and − 20 °C for 12 h. **b** GCD curves of supercapacitors with PSLM/LiCl electrolyte and PAC electrodes measured at a current density of 0.2 mA cm^−^^2^ after storage at 25 and − 20 °C for 12 h. **c** Long-term GCD cycling test of the supercapacitor with PSLM/LiCl hydrogel electrolyte and PAC electrodes conducted at a current density of 4 mA cm^−^^2^ at 25 °C (inset: GCD curves of the 1 st and 45,000th cycles). **d** Ragone plots of supercapacitors with PSLM/LiCl hydrogel electrolyte and PAC electrodes. **e** CV and **f** GCD curves of three-connected supercapacitors with PSLM/LiCl hydrogel electrolyte and PAC electrodes measured at 0.2 mA cm^-2^

To explore electrochemical performances of supercapacitors with PSLM/LiCl hydrogel electrolyte, three carbon-based electrodes such as multi-walled carbon nanotubes (MWCNTs), graphite nanofibers (GNF), and porous activated carbon (PAC) were evaluated as a working electrode for identical three-electrode conditions: silver/silver chloride as a reference electrode, platinum plate as a counter electrode in LiCl aqueous electrolyte (Fig. S10). Among them, porous activated carbon exhibited the largest CV area, and the longest GCD time; leading to highest specific capacitance of 140.75 F g^−^^1^ at a current density of 1 A g^−1^, which was significantly higher than that of graphite nanofibers (21.2 F g^−^^1^) and MWCNTs (14.5 F g^−^^1^). Follow-up tests at various scan rates and current densities (Fig. S11) showed that the electrode retained a capacitance of 60 F g^−^^1^ at 10 A g^−^^1^ of current density and the CV curves remained nearly rectangular even at 200 mV s^−^^1^, indicating excellent rate performances of porous activated carbon in LiCl aqueous electrolyte. Based on these results, porous activated carbon loaded on carbon cloth substrate was selected as the electrode material for assembling the symmetric supercapacitor with the PSLM/LiCl hydrogel electrolyte to explore the efficiency of the PSLM/LiCl hydrogel electrolyte for the energy storage behavior. The electrochemical performances of the supercapacitors at 25 °C were characterized as shown in Fig. S12. The internal resistance reaches approximately 16 Ω (Fig. S12a), which is higher than that of LiCl aqueous electrolyte (4.5 Ω) as shown in Fig. S10a; implying the sluggish ionic conductivity inside the hydrogel matrix. However, the familiar quasi-rectangular CV curves across scan rates from 5 to 200 mV s^−^^1^, and the corresponding GCD curves obtained under various current densities were nearly triangle and symmetric, indicating the electrical double layer capacitor (EDLC) storage mechanism of the devices; (Fig. S12b, c). The areal capacitance of the supercapacitor was further evaluated at various current densities at 25 °C, as summarized in Fig. S12d. As the current density increased from 0.2 to 4 mA cm⁻^2^, the capacitance gradually decreased from 93.52 to 67.6 mF cm^−^^2^, which can be attributed to the sluggish kinetics of ion diffusion at higher charge–discharge rates in the hydrogel matrix. Despite this reduction, the device retained over 65% of its initial capacitance at the highest current density of 4 mA cm^−2^, indicating good rate performance and fast ionic transport at the hydrogel-electrode interface. Moreover, the electrochemical measurements were further performed after storage at 25 and − 20 °C to assess the low-temperature performance of the assembled device. As shown in Fig. 5a, the CV curves recorded at a scan rate of 50 mV s^−^^1^ maintained a nearly rectangular shape and perfectly fitted CV areas under both conditions, suggesting well-preserved capacitive behavior and stable performances at large range of temperature. Likewise, the GCD curves measured at 0.2 mA cm^−^^2^ (Fig. 5b) exhibited highly symmetric profiles after storage at both 25 and − 20 °C for 12 h, with only a slight reduction in discharge time at − 20 °C. These results indicate that the device remains electrochemically stable under sub-zero conditions, thereby confirming the anti-freezing functionality of the PSLM/LiCl hydrogel electrolyte. Furthermore, the long-term electrochemical durability of the device was assessed by continuous charge–discharge cycling at a highest current density of 4 mA cm^−^^2^ (Fig. 5c). Remarkably, the capacitance retained 98% of its initial areal capacitance after 45,000 cycles. The GCD curves of the first and final cycles (inset) exhibited minimal shape distortion, further supporting the electrochemical durability of the devices. This level of cycling stability exceeds that of most reported hydrogel-based supercapacitors, which typically maintain performance over 10,000 to 20,000 cycles (Table 1). In addition to the excellent cycling stability at room temperature, the supercapacitor also demonstrates remarkable stability under low-temperature conditions. As shown in Fig. S13, when operated at − 20 °C, the device can stably run for more than 45,000 charge–discharge cycles without noticeable degradation, indicating that the hydrogel electrolyte maintains reliable ion transport and interfacial integrity at sub-zero temperatures. This outstanding stability is attributed to the robust ion transport pathways provided by the PSLM/LiCl hydrogel electrolyte based on the EDLC storage mechanism. The electrochemical performance of the supercapacitor was further investigated at elevated temperatures to determine its upper working limit. As shown in Fig. S14a, b, at 80 °C, larger CV curve areas and longer GCD discharge times are observed relative to room temperature, which can be attributed to enhanced ion mobility and reduced internal resistance in the hydrogel electrolyte. Elevated temperatures facilitate faster ion diffusion and more efficient charge accumulation at the electrode–electrolyte interface, thereby improving EDLC-based charge storage. Stable device operation is maintained up to 80 °C. In contrast, at 100 °C the device cannot sustain normal operation, likely due to electrolyte dehydration and degradation of interfacial stability (Fig. S14c). Figure 5d presents the Ragone plot of the device, showing a maximum areal energy density of 12.9 μWh cm^−^^2^ at a power density of 400 μW cm^−^^2^. In practical applications, increasing the operating voltage through series connection of individual supercapacitor units is required. As shown in Fig. 5e, f, three-connected supercapacitors exhibited an expanded electrochemical window up to 3 V, as confirmed by both CV and GCD measurements. Furthermore, the three-connected supercapacitors were able to stably power a commercial LED for over one minute, demonstrating their applicability under real operating conditions (Fig. S15 and Video S2).

**Table 1:** Comparison of hydrogel electrolytes and cyclic stability of supercapacitors

Materials	Conductivity (electrolyte) (S m^−1^)	Freezing Tolerance (electrolyte) (°C)	Elongation at break (electrolyte) (%)	Cyclic stability (device) (%@GCD cycles)	Refs
SPGL (SF/PVA/Gly/LiCl)	5.28	− 70	344	88.5@10,000	[44]
PSGL	4.00	− 40	294	98@10,000	[45]
PG_3_-Zn	2.5	− 80	1193	74@10,000	[46]
TNPE	0.56	− 30	378	90@10,000	[47]
HDES/CCNF	0.099	− 20	639	79@4000	[48]
PAAm-HAp	–	− 60	560	90@10,000	[49]
SA/PAAM/DMF/NaClO_4_	8.21	− 20	–	100@10,000	[50]
PAAm-PA	10.20	− 20	860	81%@5000	[51]
P(NaSS-co-MPTC) PA	7.42	− 30	–	82.0%@2000	[52]
**PSLM/LiCl**	**4.35**	**− 40**	**907**	**98@45,000**	**This work**

**Fig. 6:**
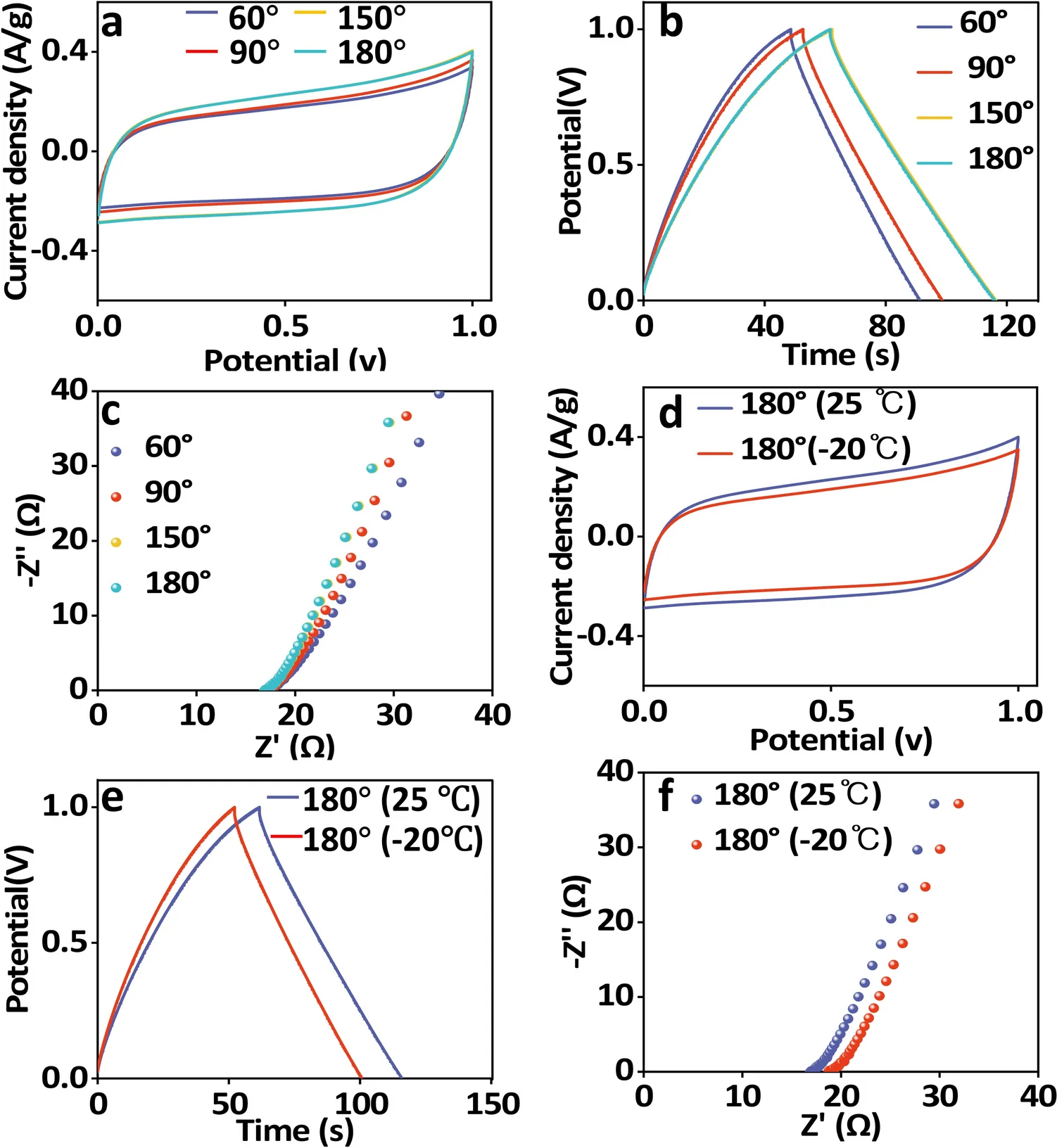
**a** CV curves of the supercapacitors measured at various bending angles. **b** GCD curves measured at various bending angles. **c** Nyquist plots of the supercapacitors recorded at various bending angles. **d** CV curves **e** GCD curves and **f** EIS spectra of the supercapacitors after storage at 25 and − 20 °C measured while deformed in 180° bending

The low-temperature adaptability of the assembled supercapacitor based on PSLM/LiCl hydrogel electrolyte was further evaluated at various bending angles. As shown in Fig. 6a, b, the supercapacitors deformed at lower bending angles (60° and 90°) exhibited the smaller CV area and the shorter GCD time than that of the supercapacitors deformed at larger bending angles (150° and 180°). As the bending angle increased, the device layers became more tightly compressed, resulting in improved interfacial contact and more stable electrochemical behavior. This trend is further confirmed by EIS measurements (Fig. 6c), where the charge-transfer resistance decreased with increasing bending angle. Moreover, three supercapacitors connected in series were able to power an LED light while being bent to 180° (Fig. S16 and Video S3), demonstrating the practical potential of the system under extreme conditions. As shown in Fig. 6d-f, the CV, GCD, and impedance responses of the supercapacitors after storage at 25 and − 20 °C show almost identical values when they are deformed in 180° bending, indicating excellent low-temperature operability even under severe mechanical deformations of the devices.

As shown in Table 1, the PSLM/LiCl hydrogel electrolytes developed in this work exhibit a high ionic conductivity of ~ 4.35 S m^−^^1^, excellent mechanical deformability (elongation at break of 907%), and reliable antifreeze property down to − 40 °C. More importantly, the assembled supercapacitor demonstrates outstanding cycling durability, maintaining 98% capacitance retention over 45,000 GCD cycles, which surpasses most literature-reported values. These results underscore the potential of the PSLM/LiCl hydrogel electrolytes for use in next-generation flexible and low-temperature energy storage devices.

## Conclusions

In this work, we developed ultra-stretchable and anti-freezing hydrogels cross-linked by liquid metal particle initiators for applications in stretchable and soft supercapacitors. The hydrogel was rapidly cross-linked via the free-radical polymerization using the liquid metal nanoparticles. During polymerization of the hydrogel, stearyl methacrylate was introduced to form hydrophobic associations within the polymer network, thereby maximizing the physical cross-linking density of the hydrogel, resulting in ultra-stretchability (907%). After polymerized, the hydrogel was immersed in DI water with 30 wt% of LiCl to achieve excellent ionic conductivity (~ 4.35 S m^−^^1^ at 25 °C) and disrupt hydrogen bonding in the polymeric network, thereby lowering the freezing point and imparting stable mechanical flexibility and electrical behavior even at sub-zero temperatures (3.39 S m^−^^1^ at − 20 °C). The symmetric supercapacitor fabricated by using the hydrogel electrolyte and porous activated carbon electrodes achieved a high areal capacitance of 93.52 mF cm^−^^2^, an energy density of 12.9 µWh cm^−^^2^, and retained 98% of its capacitance after 45,000 cycles at 4 mA cm^−^^2^. Additionally, the supercapacitor exhibited stable energy storage behavior at both ambient and sub-zero temperature while mechanically deformed, as confirmed by both electrochemical tests and powering of LEDs. The hydrogel electrolytes developed in this work exhibiting ultra-stretchability, anti-freezing ability and excellent ionic conductivity would be promising for next-generation soft, deformable, and cold-tolerant energy storage devices.

## Supplementary Information

Below is the link to the electronic supplementary material.

Supplementary Material 1

Supplementary Material 2

Supplementary Material 3

Supplementary Material 4
