# Supplementary material for: Ultra-Stretchable Anti-Freezing Hydrogel Electrolytes Cross-Linked by Liquid Metal Particle Initiators Toward Soft Energy Storage Devices
Source: Nanomicro Lett. 2026 Mar 13;18:283. doi: 10.1007/s40820-026-02126-7 (PMC12988136; doi:10.1007/s40820-026-02126-7)
Supplement: Supplementary file 1 — Supplementary Material 1 [file 40820_2026_2126_MOESM1_ESM.docx]

Supporting Information for

**Ultrastretchable Anti-Freezing Hydrogel Electrolytes Cross-Linked by Liquid Metal Particle Initiators Toward Soft Energy Storage Devices**

Qingshi Zhang^1^, Priyanuj Bhuyan^2^, Que Thi Nguyen^1^, Xia Sun^3^, Kunlong Liang^1^, Mukesh Singh^1^, Subir Kumar Pati^1^, Xianglan Li^1^, Yeeshu Kumar^1^ and Sungjune Park^1,2*^

^1^ School of Chemical Engineering, Sungkyunkwan University (SKKU), Suwon 16419, Republic of Korea

^2^ Wearable Fluidic, Inc, Suwon, 16419, Republic of Korea

^3^ Sustainable Functional Biomaterials Laboratory, Bioproducts Institute, Department of Wood Science, Faculty of Forestry, University of British Columbia, Vancouver, BC, V6T 1Z4 Canada

*Corresponding author. E-mail: [sungjunepark@skku.edu](mailto:sungjunepark@skku.edu) (Sungjune Park)

**Supplementary Figures and Tables**


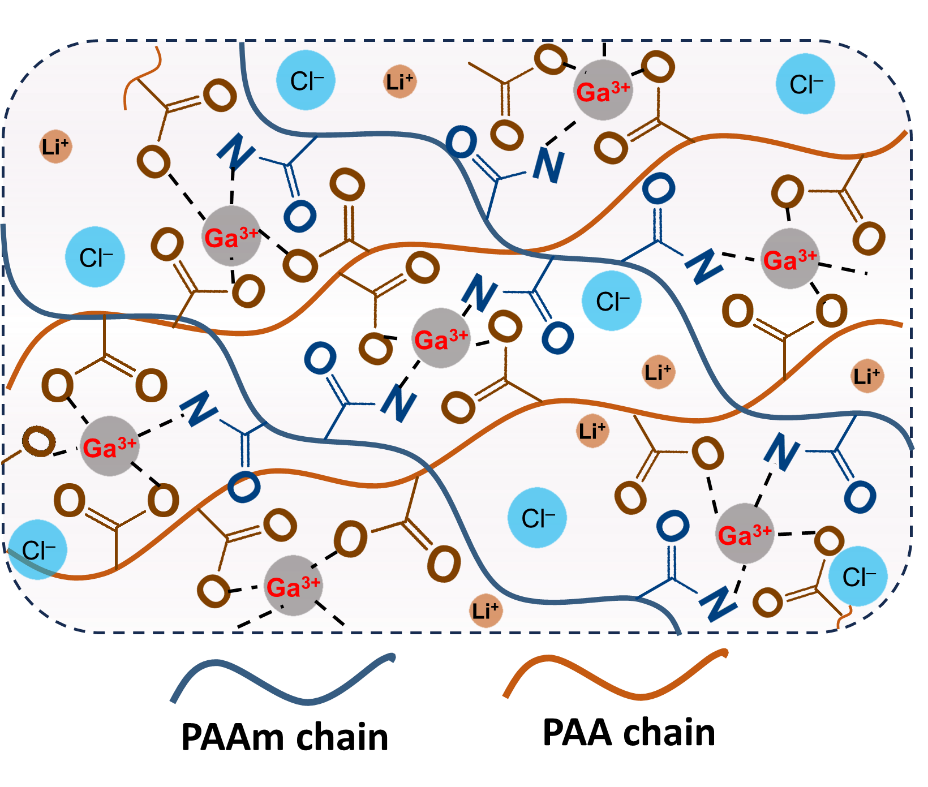


**Fig. S1** Schematic illustration of ionic crosslinking between Ga³⁺ ions and carboxylate groups on poly(acrylic acid) chains

**
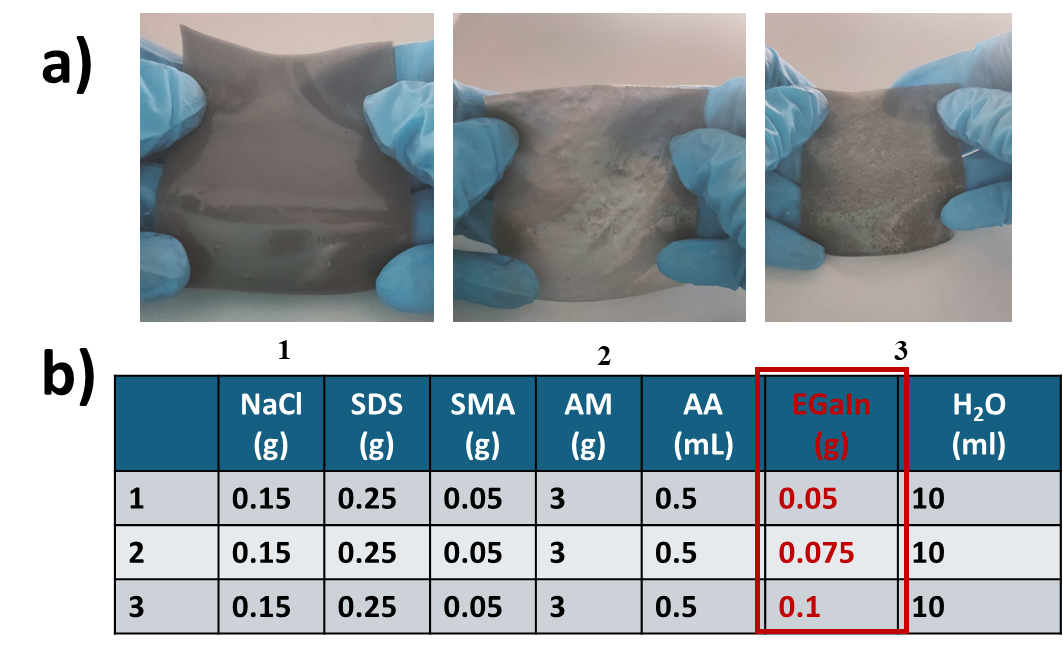
**

**Fig. S2 (a**) Photographs of the hydrogels synthesized by using different liquid metal contents (0.05 g, 0.075 g, and 0.1 g). (**b**) The amount of each component for the hydrogels


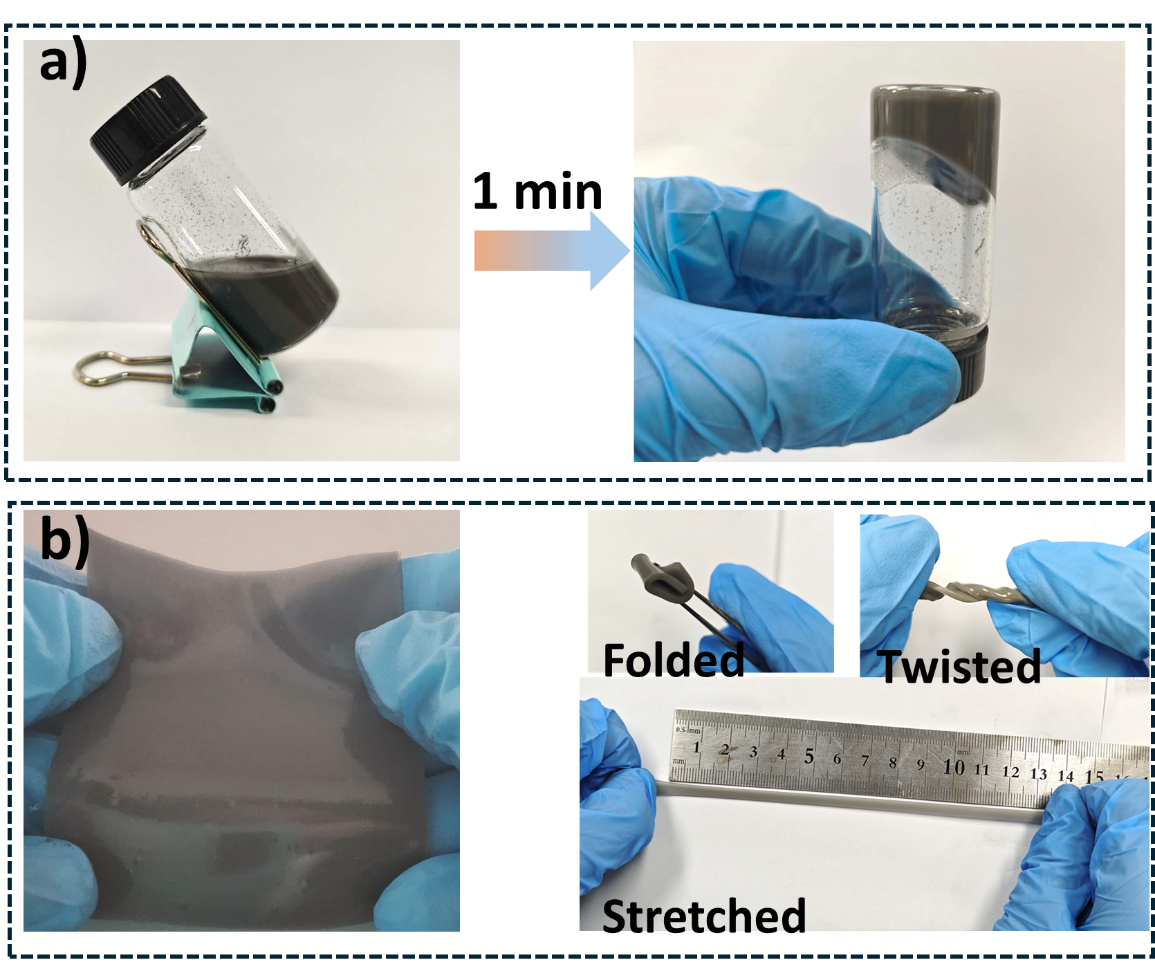


**Fig. S3** (**a**) Photos showing rapid polymerization of PSLM hydrogel. (**b**) Photos showing deformability of PSLM hydrogel

**
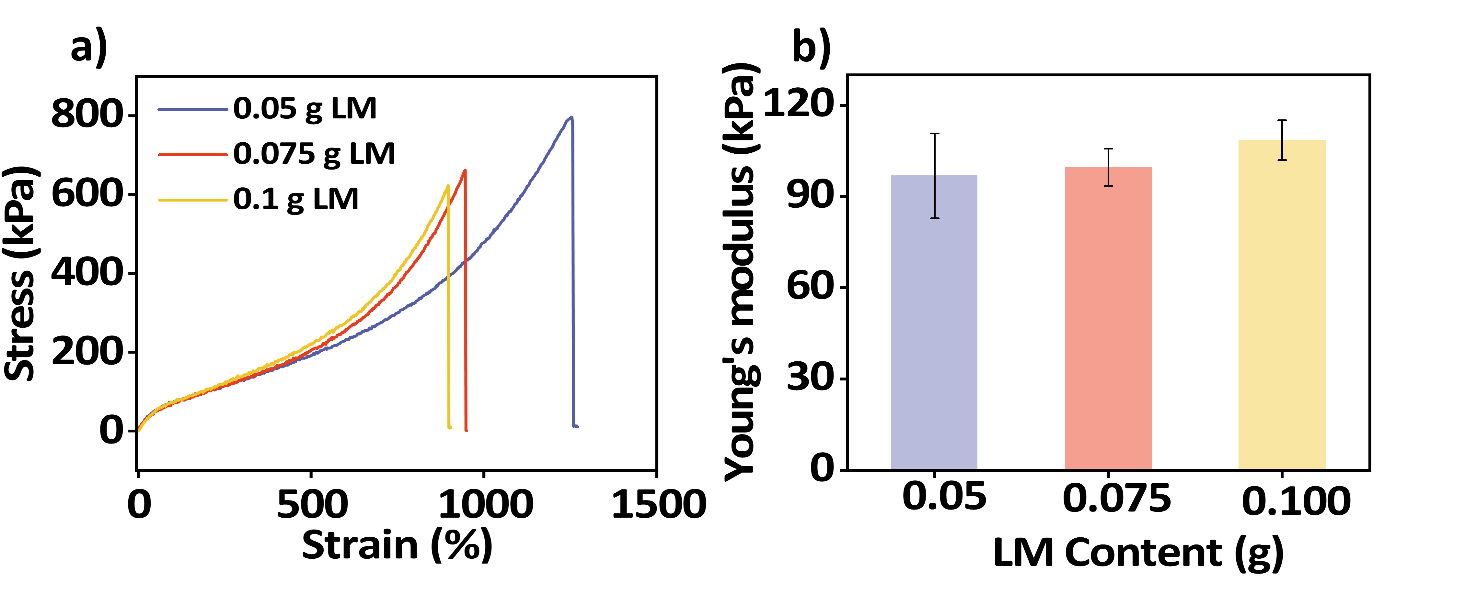
**

**Fig. S4** (**a**) Tensile stress–strain curves and (**b**) Young's modulus of hydrogels containing different content of LM

**
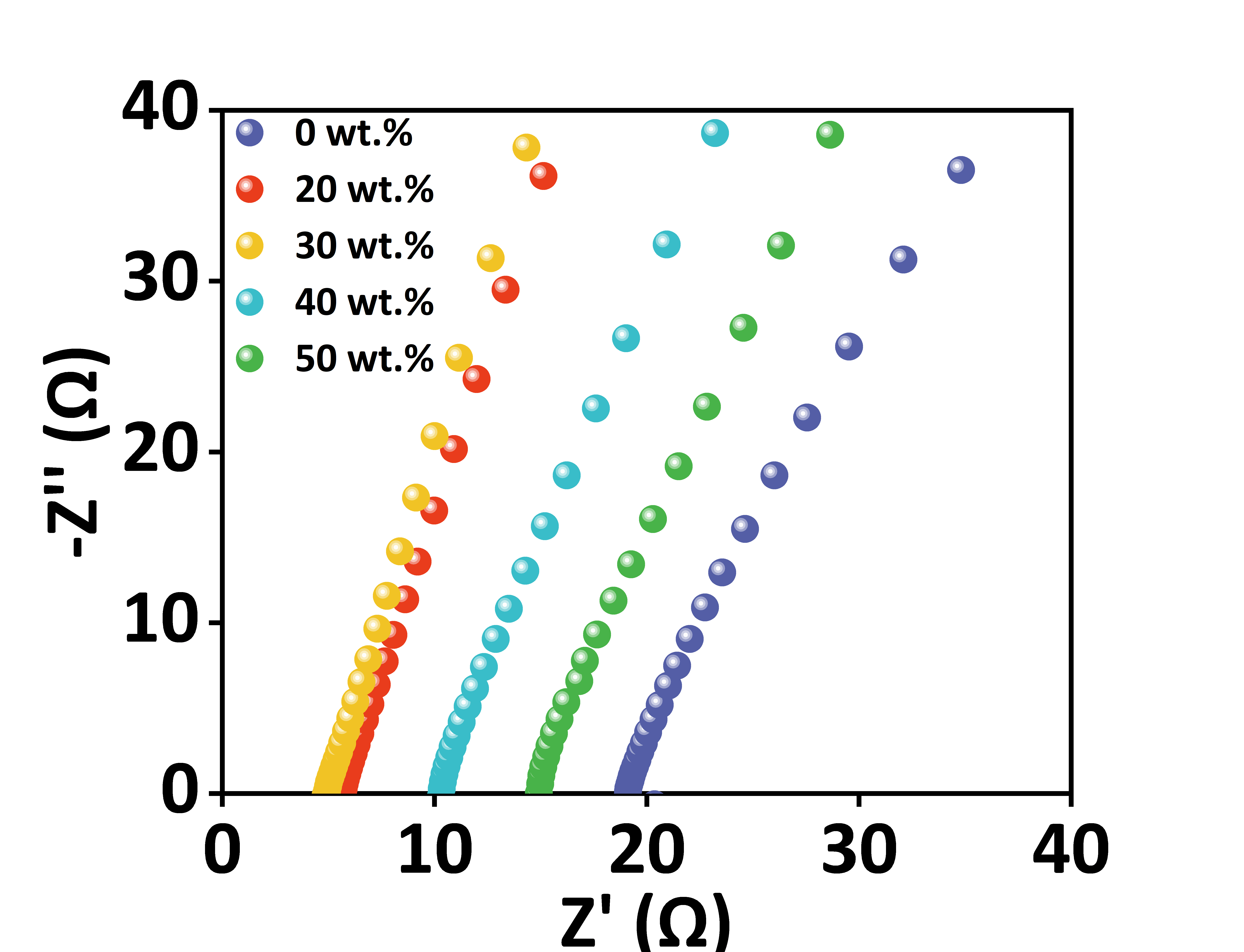
**

**Fig. S5** Nyquist plots of the hydrogel after immersing in DI water containing various concentrations of LiCl


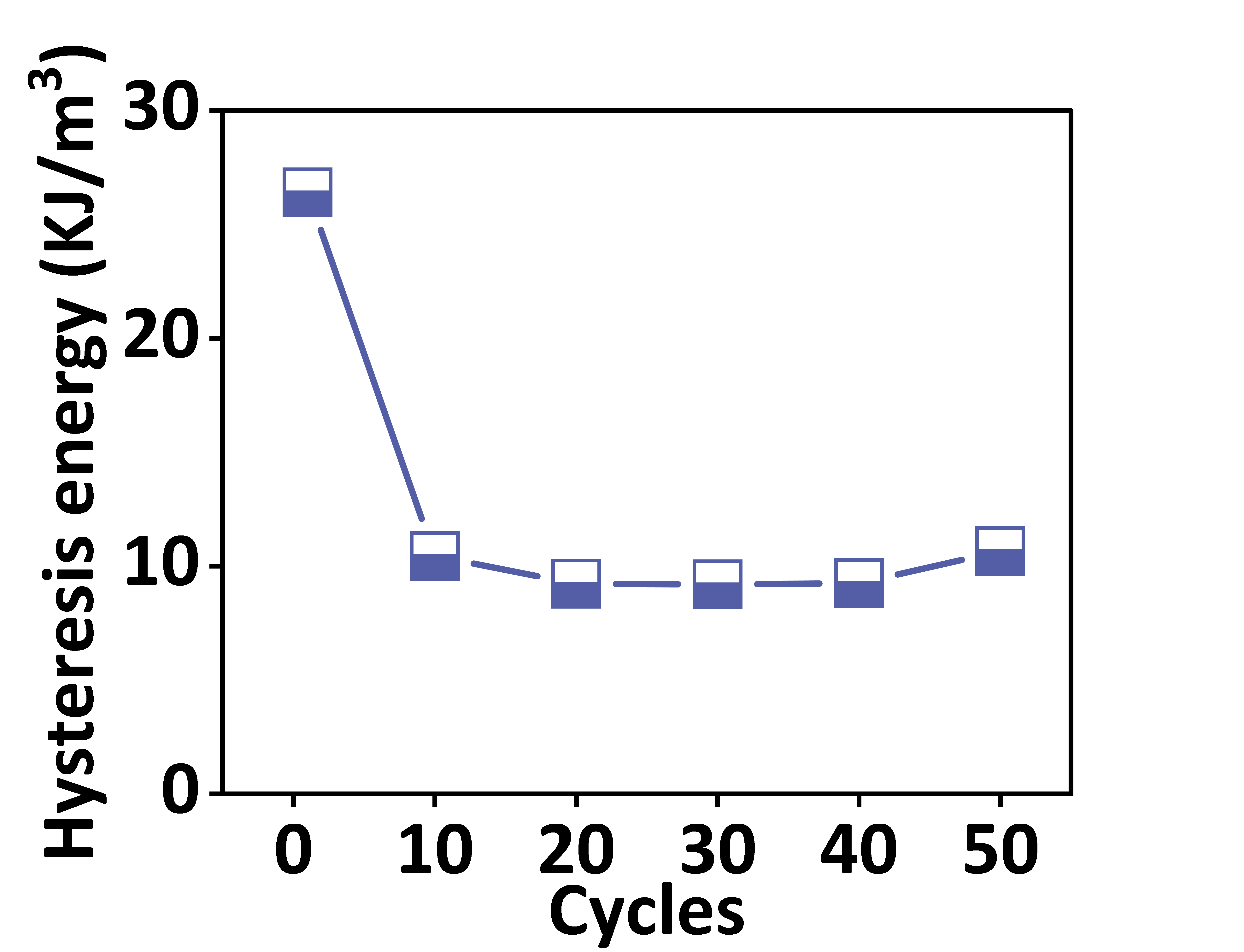


**Fig. S6** Hysteresis energy of PSLM/LiCl hydrogels during each cycle of tensile strains with a maximum strain of 200%

**
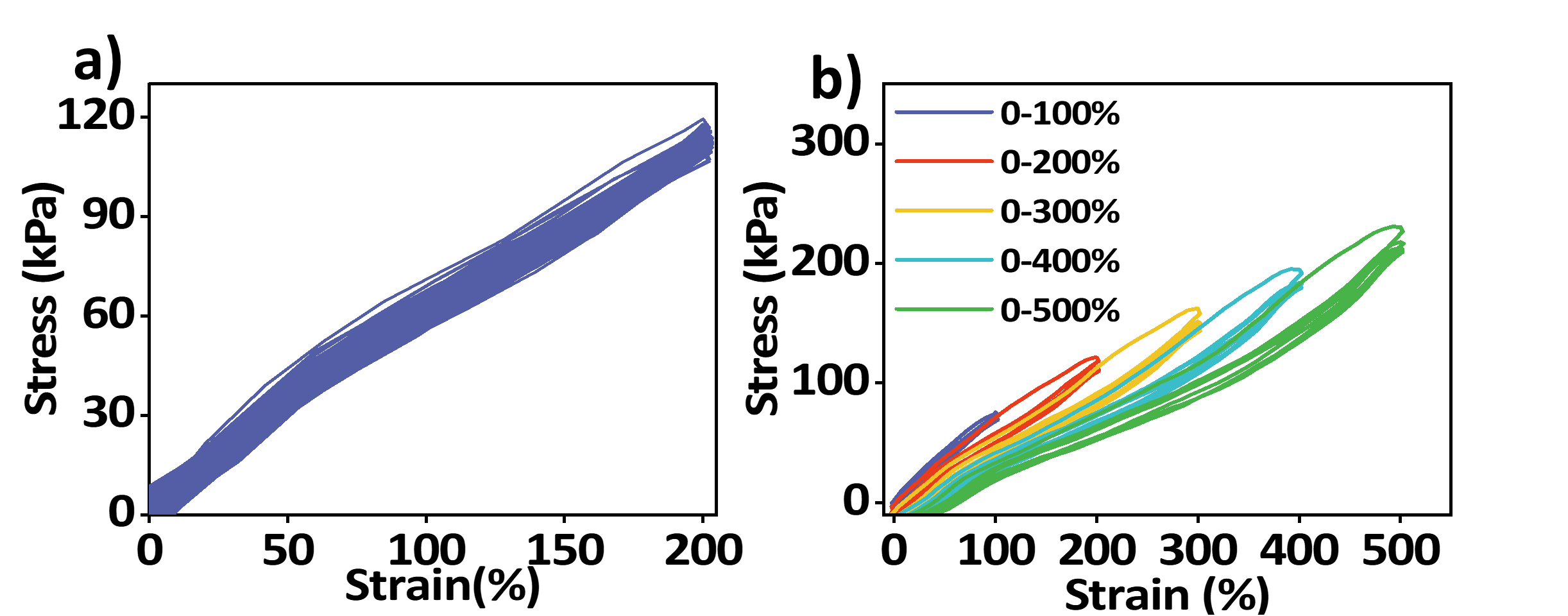
**

**Fig. S7 (a)** Stress–strain curves for PSLM/LiCl hydrogel during 50 consecutive tensile strains until a maximum strain of 200% after storage at –20 °C for 12h. (**b**) Stress–strain curves for PSLM/LiCl hydrogel during 4 cyclic tensile strains until various maximum strains after storage at –20 °C for 12 h

**
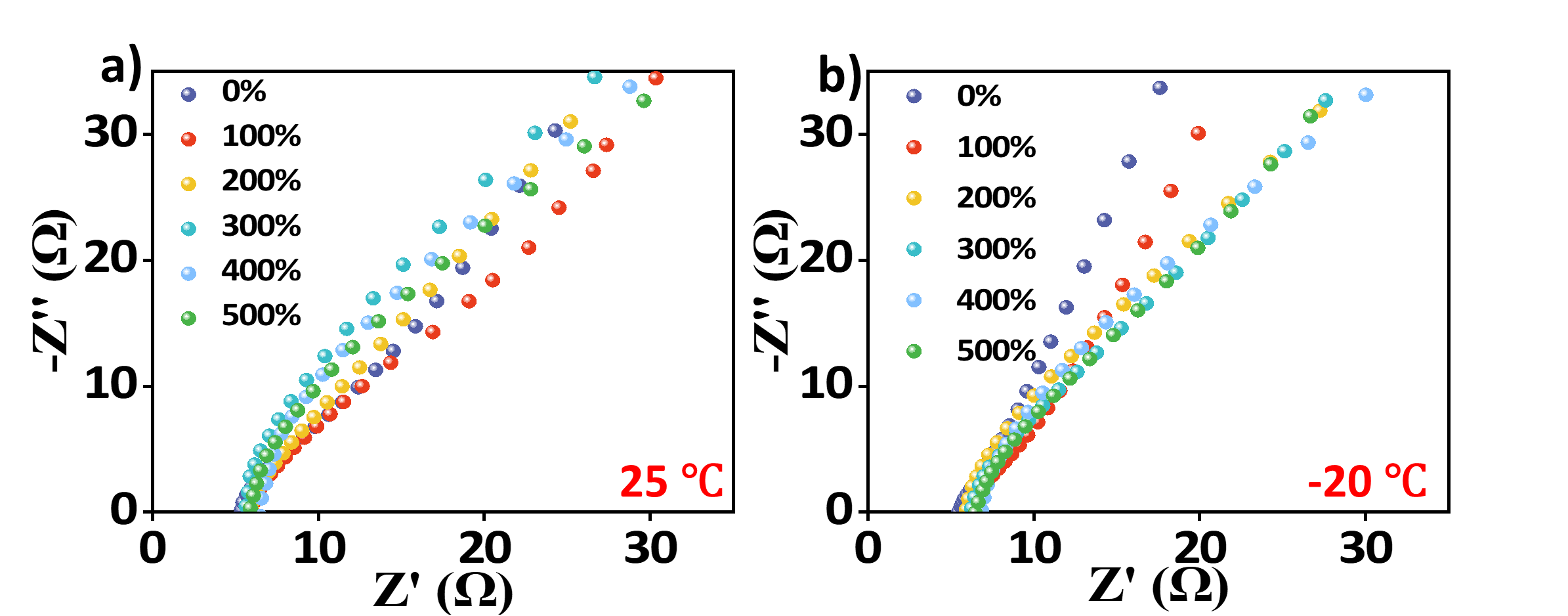
**

**Fig. S8** Nyquist plots of hydrogels in different stretching states after storage at (**a**) 25 °C and (**b**) -20 °C

**
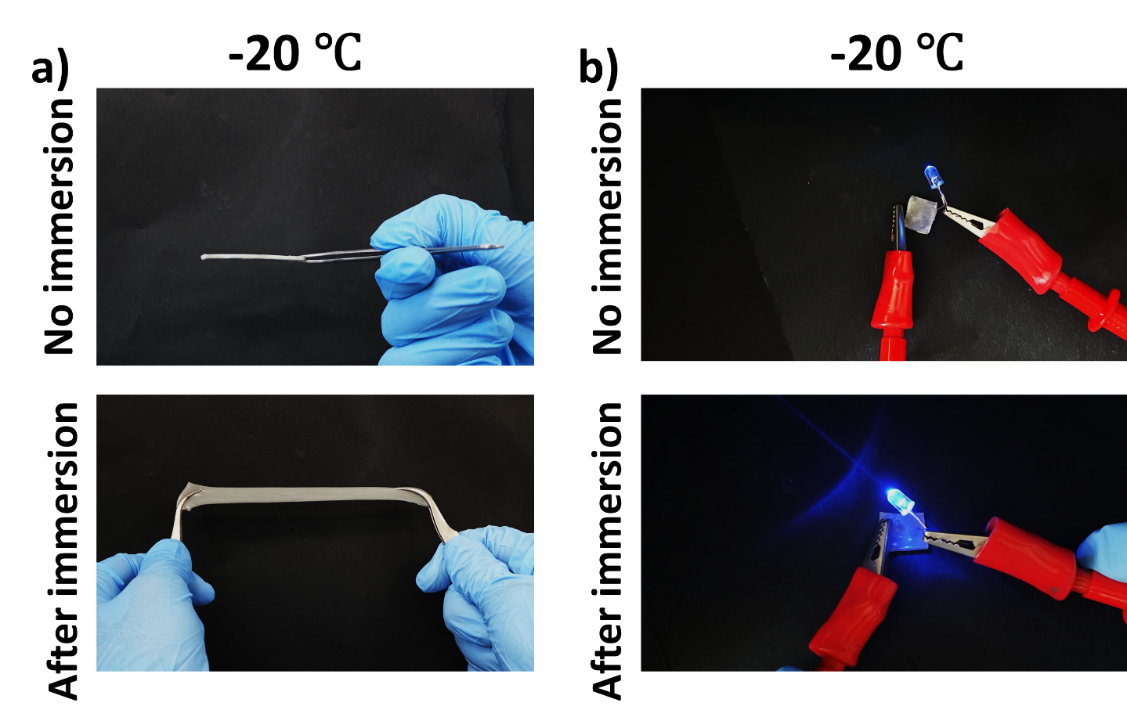
**

**Fig. S9** (**a**) Photographs of hydrogels immersed and non-immersed in DI water containing 30 wt.% of LiCl after storage at −20 °C for 12 h, respectively. Non-immersed hydrogel exhibits stiff state and immersed hydrogel shows stretchability after storage at −20 °C for 12 h, respectively. (**b**) LED lighting demonstration using hydrogels immersed and non-immersed in DI water containing 30 wt.% of LiCl after storage at −20 °C for 12 h, serving as ionic conductors

**
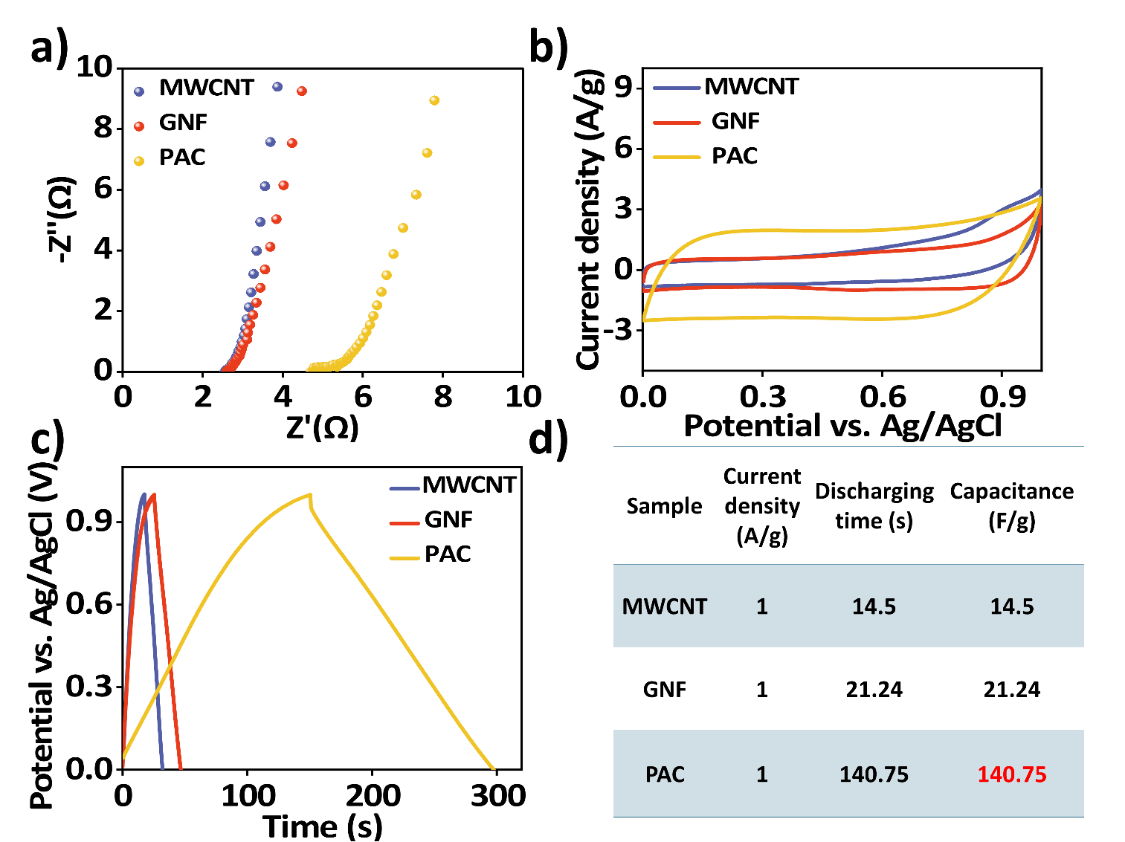
**

**Fig. S10** The electrochemical characteristics of three electrode systems using MWCNT, graphite nanofiber (GNF) and porous activated carbon (PAC) electrodes as a working electrode with PSLM/LiCl hydrogel electrolyte, respectively: (**a**) Nyquist plots, (**b**) CV curves measured at 50 mV/s of scan rate, (**c**) GCD curves measured at 1 A/g of current density and (**d**) Specific capacitance calculations

**
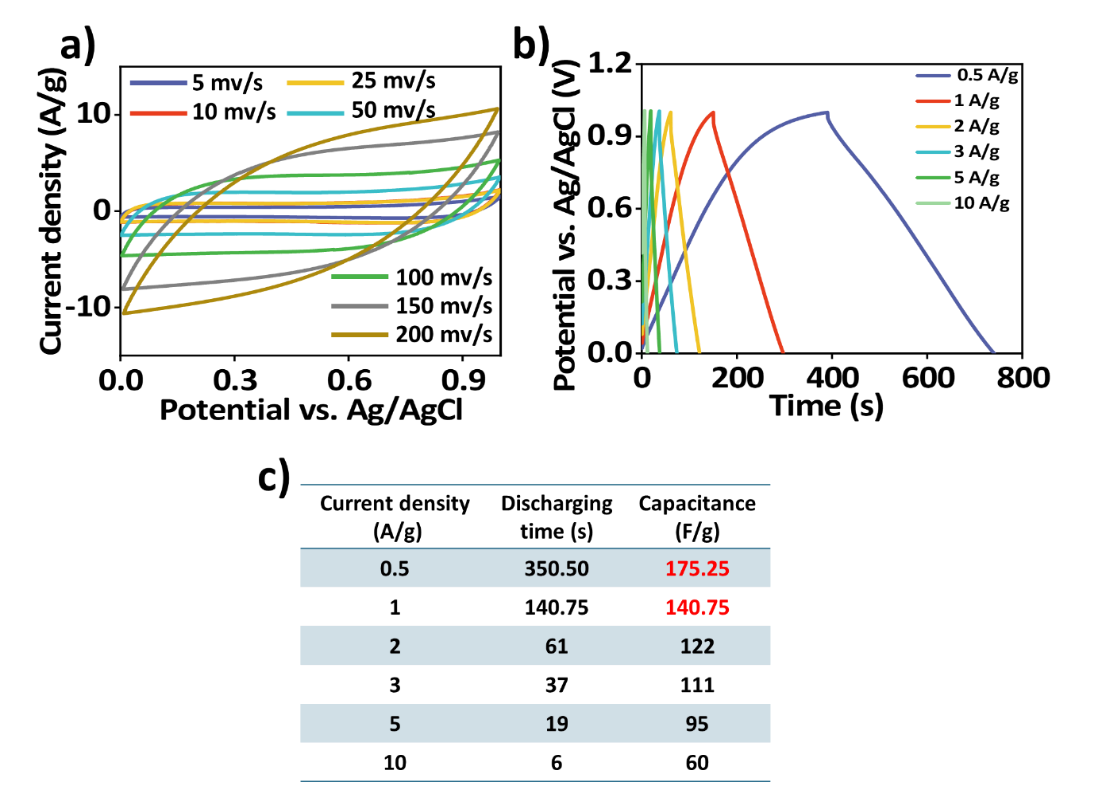
**

**Fig. S11** The performances of three-electrode systems using porous activated carbon electrodes at various conditions: (**a**) CV curves measured at different scan rates (5 – 200 mV/s), (**b**) GCD curves measured at different current densities (0.5–10 A/g), and (**c**) the corresponding specific capacitances calculated viz. GCD results

**
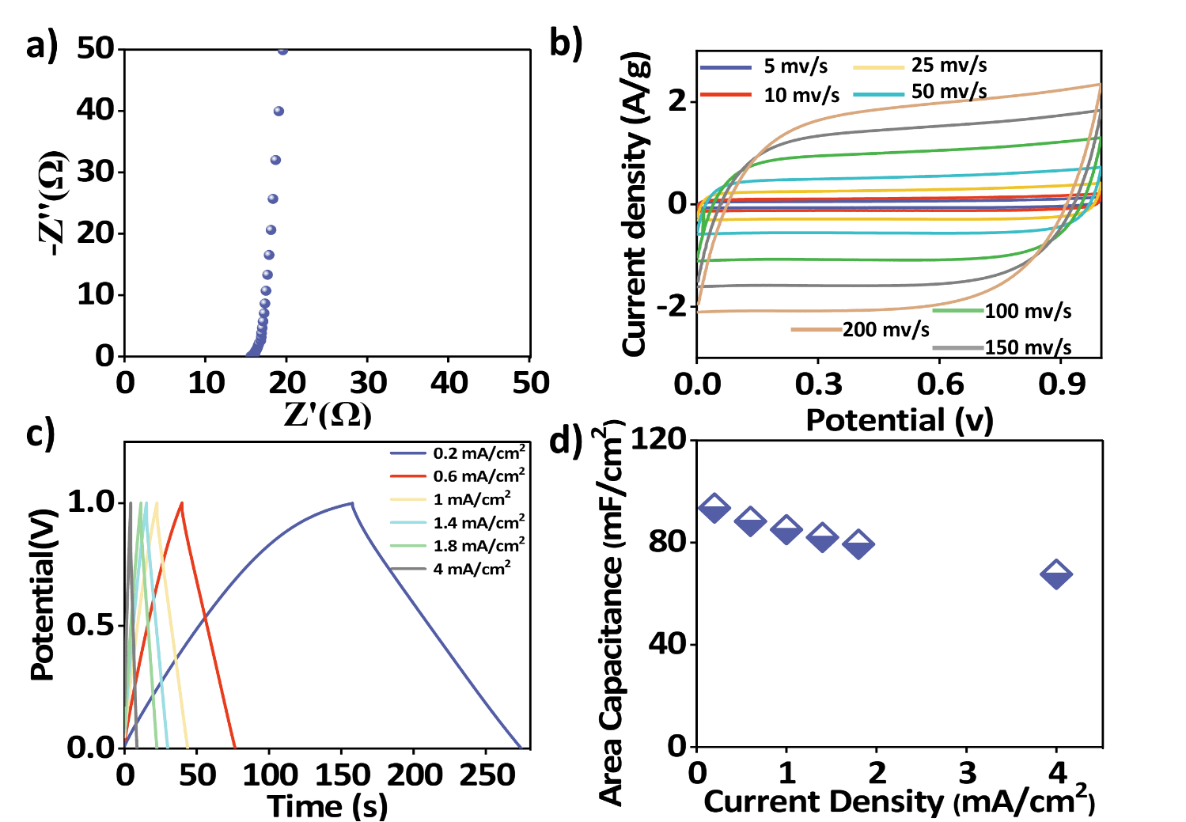
**

**Fig. S12** Performances of supercapacitors with porous activated carbon electrodes and PSLM/LiCl hydrogel electrolyte: (**a**) Nyquist plots, (**b**) CV curves measured at different scan rates (5 – 200 mV/s), (**c**) GCD curves measured at different current densities (0.2 – 4 mA/cm^2^) and (**d**) Areal capacitance of the supercapacitor with PSLM/LiCl hydrogel electrolyte at various current densities


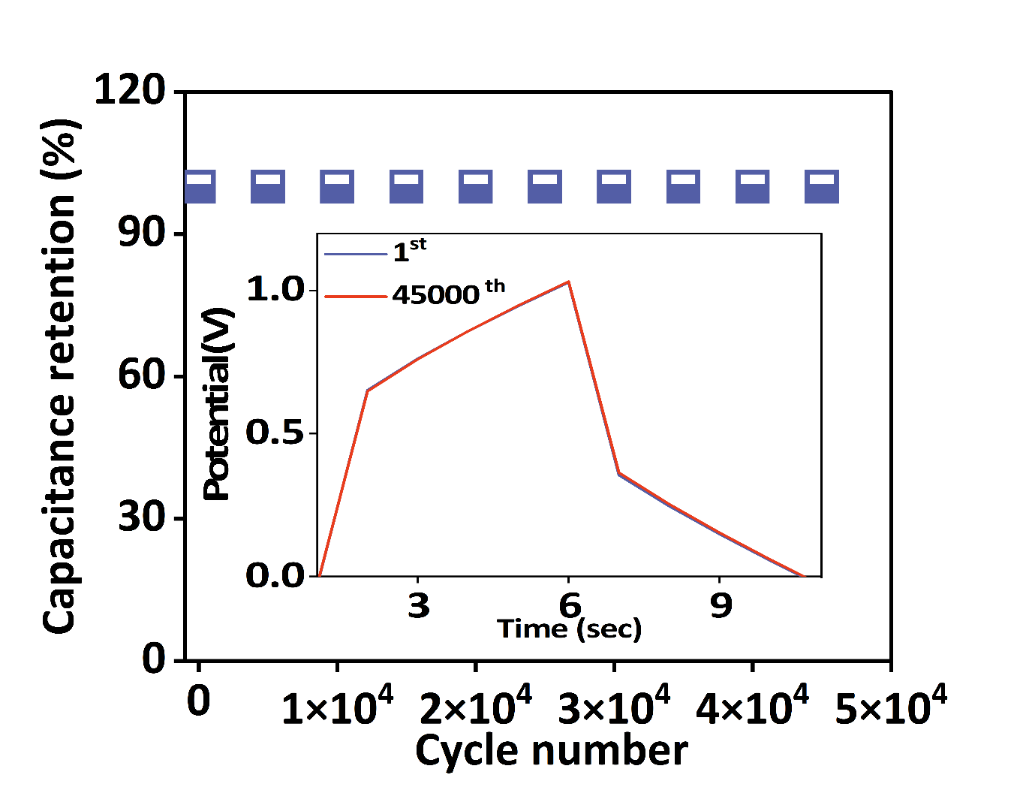


**Fig. S13** Long-term GCD cycling test of the supercapacitor with PSLM/LiCl hydrogel electrolyte and PAC electrodes conducted at a current density of 4 mA cm⁻² at -20 °C (inset: GCD curves of the 1^st^ and 45,000^th^ cycles)


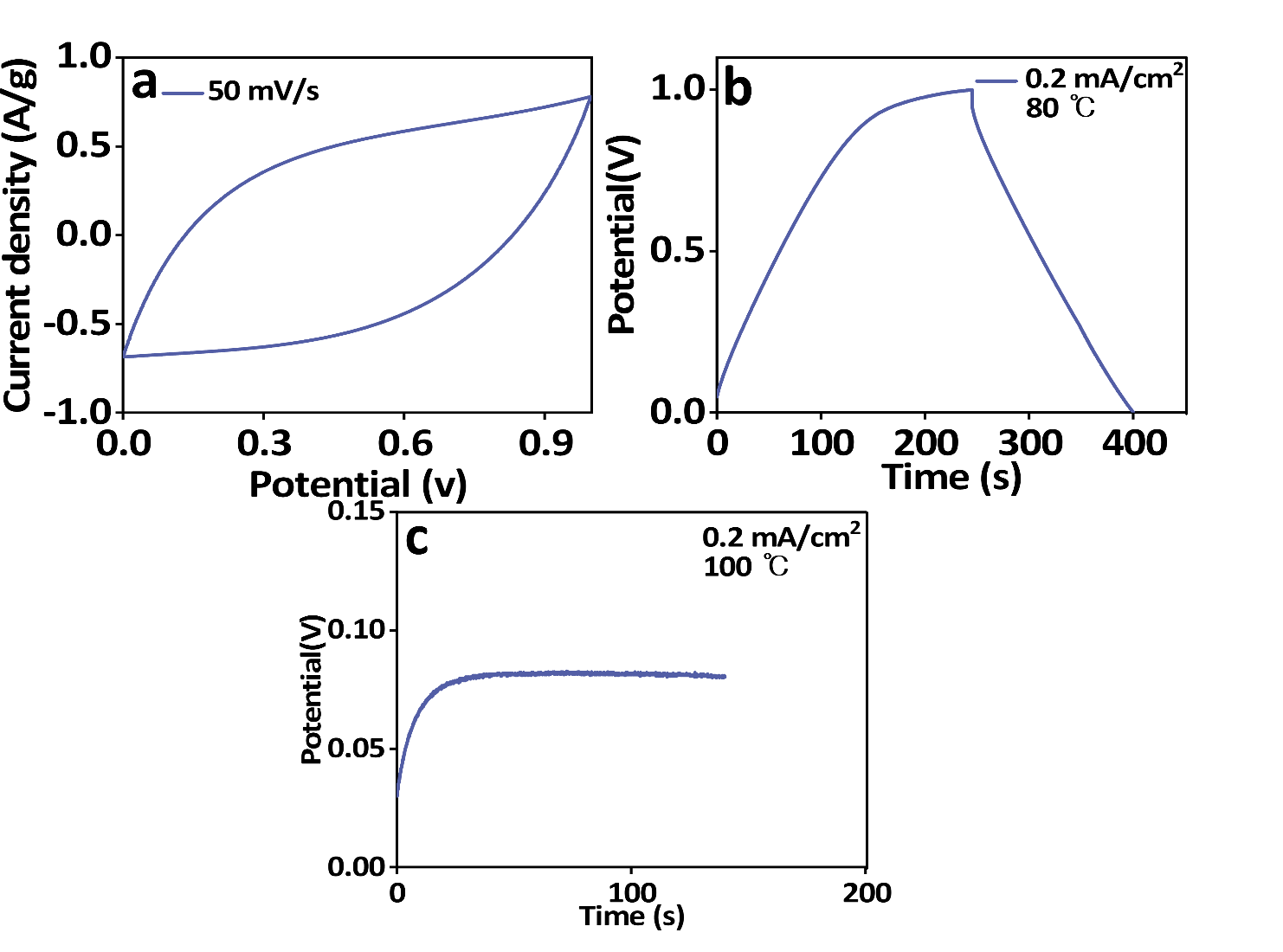


**Fig. S14** (**a**) CV curves measured at 80 °C at a scan rate of 50 mV s⁻¹, (**b**) GCD curves measured at 80 °C at 0.2mA/cm^2^ and (**c**) GCD curves measured at 100 °C under the same current density

**
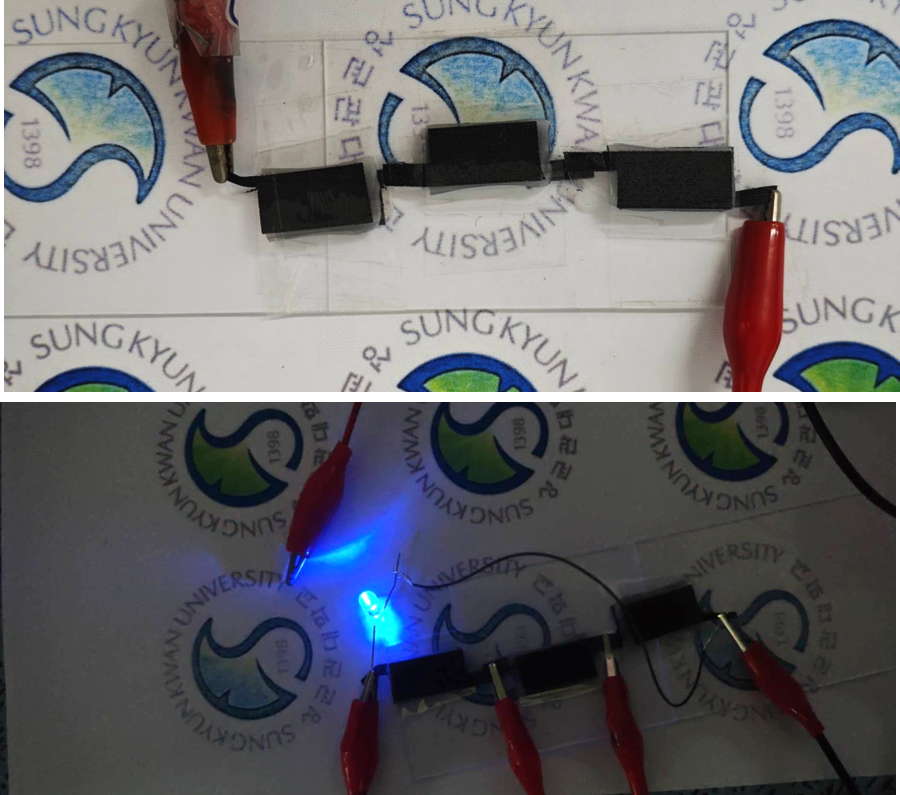
**

**Fig. S15** Photograph of an LED light powered by three supercapacitors connected in series


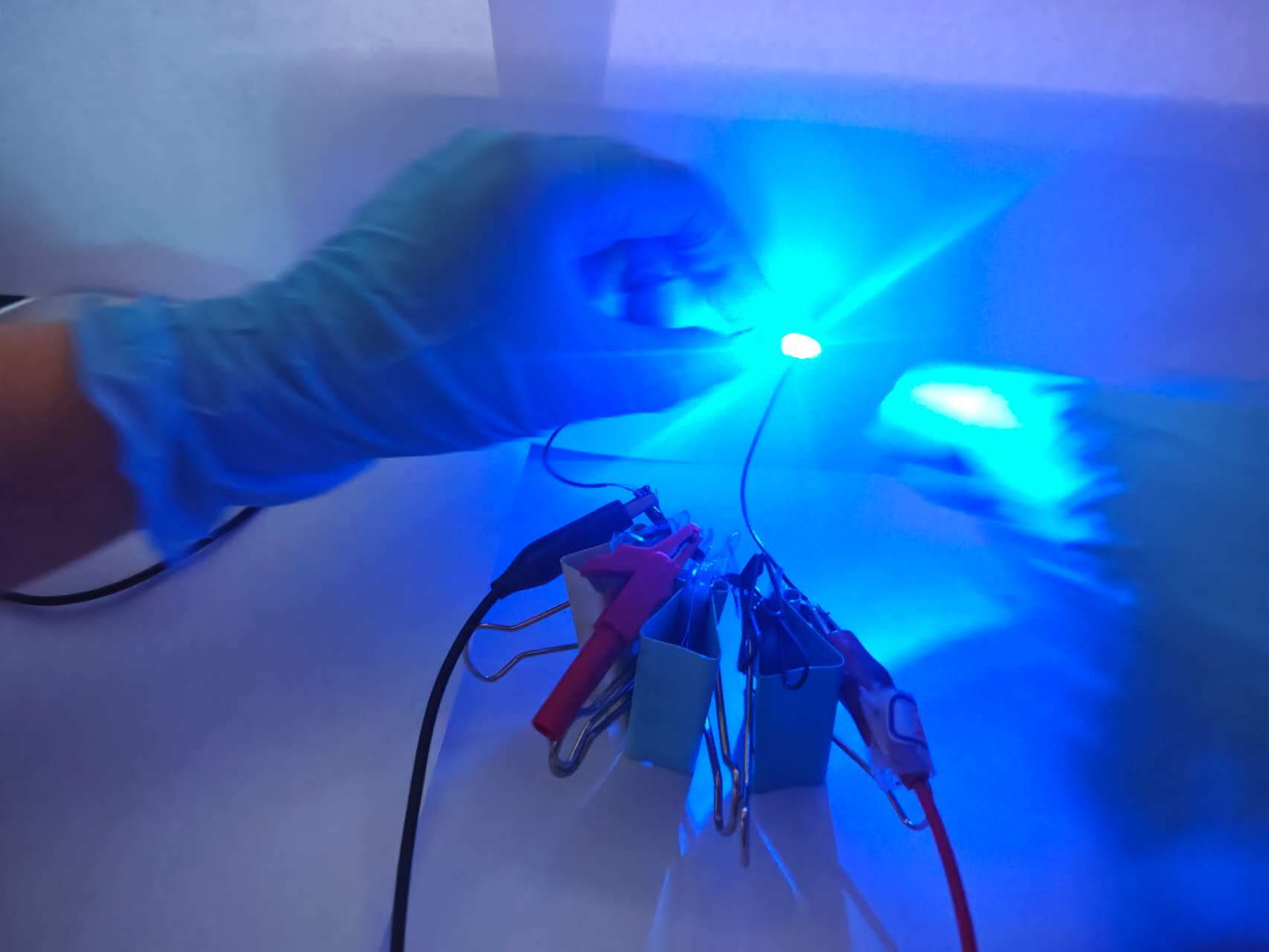


**Fig. S16** Photograph of three-connected supercapacitors powering an LED light in 180° bend
